# Dihydromyricetin preserves β-cell function in type 1 diabetes via PI3K/AKT-mediated metabolic reprogramming

**DOI:** 10.3389/fnut.2025.1682308

**Published:** 2025-10-02

**Authors:** Jia Li, Lijia Li, Tahui Lin, Houtan Huang, Jie Ren, Jengyuan Yao

**Affiliations:** ^1^School of Public Health, Fujian Medical University, Fuzhou, Fujian, China; ^2^Key Laboratory of Functional and Clinical Translational Medicine, Xiamen Medical College, Universities of Fujian Province, Xiamen, Fujian, China

**Keywords:** dihydromyricetin, vine tea, nutraceutical, metabolomics, type 1 diabetes

## Abstract

**Background:**

Food-derived flavonoids are emerging as nutraceutical agents for glycemic control. Dihydromyricetin (DMY), the signature flavanonol of vine tea (*Ampelopsis grossedentata*), has long been consumed in South China, yet its antidiabetic potential remains underexplored.

**Methods:**

We administered DMY (50 and 100 mg/kg/day, 12 days) to streptozotocin-induced type 1 diabetic mice. Fasting glycemia, lipid panels, and HOMA-β were evaluated 4 weeks after DMY administration. Untargeted UPLC-QTOF metabolomics combined with network pharmacology pinpointed pathway hubs, while experiments in INS-1 β-cells using the PI3K inhibitor LY294002 verified the pathway’s involvement.

**Results:**

DMY reduced hyperglycemia, corrected dyslipidemia, and preserved islet architecture. Metabolomics indicated a shift toward a normal plasma profile, with the arachidonic acid, linoleic acid, and steroid hormone pathways being the most responsive. Six hub targets (PTGS2, IL6, AKT1, IL1B, BCL2, CASP3) mapped to eicosanoid signaling, apoptosis, and PI3K/AKT axis. Docking and cell assays confirmed direct binding and PI3K/AKT-dependent cytoprotection, evidenced by restored p-AKT, lowered ROS, and reduced caspase-3 cleavage.

**Conclusion:**

DMY, a readily accessible food-derived bioactive compound, reprograms lipid-inflammatory metabolism and activates PI3K/AKT to safeguard β-cell viability, highlighting its nutraceutical promise for dietary management of autoimmune diabetes.

## Introduction

Type 1 diabetes mellitus (T1DM) is a chronic autoimmune disease affecting nearly 10 million individuals worldwide, with an increasing global incidence (1). It is characterized by immune-mediated destruction of pancreatic β-cells, primarily triggered by autoreactive T and B lymphocytes (2). Due to their intrinsically low expression of antioxidant enzymes such as catalase and glutathione peroxidase, β-cells are particularly susceptible to oxidative stress (3). Excessive reactive oxygen species (ROS) promote endoplasmic reticulum and mitochondrial dysfunction, amplify pro-inflammatory cytokine production, and accelerate β-cell apoptosis, thereby driving disease progression (4).

Although exogenous insulin remains the cornerstone of T1DM management, it neither halts autoimmune β-cell destruction nor preserves residual β-cell mass. Immunotherapies such as anti-CD3 monoclonal antibodies (e.g., teplizumab) can delay disease onset but show limited efficacy and may cause immune-related adverse effects (5). These challenges highlight the need for adjunctive therapies that are both effective and safe. Flavonoids—plant-derived polyphenolic compounds—have gained attention for their antioxidant, anti-inflammatory, and glucose-lowering properties in diabetic models (6). Notably, compounds such as quercetin and kaempferol protect β-cells by activating PI3K/AKT signaling and enhancing the expression of anti-apoptotic proteins including BCL-2 (7).

Dihydromyricetin (DMY, also known as ampelopsin) is the major flavanonol in *Ampelopsis grossedentata* (vine tea), with six hydroxyl groups conferring potent free radical–scavenging activity (8). In this study, we used a chemically synthesized DMY monomer (≥98% HPLC) that is structurally identical to the vine-tea flavanonol, to avoid batch variability inherent to plant extracts and to enable mechanism-focused assays. Preclinical studies have shown that DMY improves glucose and lipid metabolism, reduces insulin resistance, and alleviates hepatic steatosis in high-fat diet and non-alcoholic fatty liver disease models (9, 10). It also attenuates diabetic renal fibrosis and modulates gut-derived incretin secretion, underscoring its systemic metabolic regulatory potential (11, 12). However, its efficacy in T1DM remains largely unexplored, and the molecular mechanisms underlying its potential β-cell protective actions are poorly defined. In particular, no study has systematically combined untargeted metabolomics and network pharmacology to delineate the metabolic and signaling pathways modulated by DMY in autoimmune diabetes.

To address this gap, we investigated the protective effects of DMY in streptozotocin-induced T1DM mice using a multi-omics strategy. Plasma metabolomics and network pharmacology were integrated to identify DMY-regulated pathways and targets. Protein–protein interaction (PPI) networks and molecular docking were used to pinpoint core targets, while functional validation in INS-1 β-cells treated with a PI3K inhibitor (LY294002) confirmed the mechanistic involvement of the PI3K/AKT pathway. This combined *in vivo*, *in vitro*, and in silico approach provides new insights into the antioxidant and antidiabetic potential of DMY as a candidate adjunctive therapy for T1DM.

## Materials and methods

### Chemicals and reagents

Dihydromyricetin (DMY; chemically synthesized monomer, ≥98% HPLC; Aladdin, Shanghai, China; Cat# D299476) and streptozotocin (STZ; Macklin, Shanghai, China; Cat# S817944) were used. Dexamethasone (DXM), glycated hemoglobin (HbA1c) kits, and general biochemical reagents were obtained from Solarbio (Beijing, China) and Smart (Chengdu, China). ELISA kits for insulin, HDL-C, and LDL-C were from Jiancheng Bioengineering Institute (Nanjing, China). For cell experiments, the PI3K inhibitor LY294002 (MedChemExpress, NJ, USA; Cat# HY-10108), CCK-8 kit (GLPBIO, Guangzhou, China; Cat# GK10001), ROS assay kit (Beyotime, Shanghai, China; Cat# S0033S), antifade mounting medium with DAPI (Pusiteng, Shanghai, China; Cat# PS1165), and 2,2,2-tribromoethanol (Aladdin; Cat# T161626) were used. Primary antibodies against HO-1, SOD1, COX2, IL6, IL1B, AKT, p-AKT, BCL2, CASPASE3, cleaved-CASPASE3, PI3K, p-PI3K, P65, p-P65, PTGER2, and β-actin were sourced from Abcam, HUABIO, Affinity, ZENBIO, Santa Cruz, and Proteintech. INS-1 cells were purchased from the National Infrastructure of Cell Line Resource (Beijing, China).

### Animal experiments

Male C57BL/6 mice (6–8 weeks, 20 ± 2 g; SPF grade) were supplied by Fuzhou Nuoton Biotechnology Co., Ltd. (SCXK (ZHE) 2019-0002). Mice were acclimated for 2 weeks at 18–26 °C (40–60% humidity, 12 h light/dark) with free access to chow and water. All protocols were approved by Xiamen Medical College Ethics Committee (No. 20240207015) and followed ARRIVE guidelines (Animal Research: Reporting of *In Vivo* Experiments). Mice were randomized into five groups: Normal (*n* = 12), T1DM model (*n* = 10), DMY50 (50 mg/kg, *n* = 10), DMY100 (100 mg/kg, *n* = 10), and pharmacological comparator DXM (dexamethasone, 1 mg/kg, *n* = 10). Except Normal, mice received intraperitoneal STZ (50 mg/kg) once daily for 5 consecutive days after a 9 h fast. STZ was freshly prepared in ice-cold sterile PBS (pH 7.4), protected from light, and injected within 10 min of dissolution; any remaining solution was discarded after 15 min. PBS was selected instead of citrate buffer to minimize additional acidity and avoid confounding pancreatic injury, a strategy also reported in previous STZ protocols. Successful induction was defined as fasting blood glucose > 11.1 mmol/L on day 7. DMY or DXM was administered i.p. once daily for 7 days before and 5 days during STZ injection. Normal and T1DM groups received PBS. Thereafter, mice remained on standard diet for 4 weeks.

At the experimental endpoint, mice were fasted overnight and deeply anesthetized with tribromoethanol (250 mg/kg, i.p.). While under a surgical plane of anesthesia (no pedal reflex), animals were euthanized by exsanguination via cardiac puncture, immediately followed by cervical dislocation as a secondary physical method to ensure death. Death was confirmed by the absence of heartbeat and respiration before tissue collection. All efforts were made to minimize animal suffering.

### Physiological and biochemical measurements

Body weight, food/water intake and fasting blood glucose were recorded weekly, spanning the 7-day pre-treatment and the 4-week post-STZ period. Plasma was obtained by cardiac puncture and centrifugation (1,000 rpm, 10 min, 4 °C). Glucose (GLU), total cholesterol (TC), triglycerides (TG), HDL-C, LDL-C and HbA1c were measured using commercial kits. Insulin was quantified by ELISA; HOMA-β was calculated as [20 × insulin (μU/mL)]/[FBG (mmol/L) − 3.5]. Pancreatic injury markers (amylase, lipase, LDH, CK) were assessed via colorimetric assays.

### Histological assessment

Pancreata were fixed in 4% paraformaldehyde, paraffin-embedded, sectioned at 3 μm, and stained with H&E. Three equidistant sections per pancreas were analyzed at predefined anatomical landmarks; within predefined fields, all islets exceeding a minimum area threshold were segmented and quantified in ImageJ. Two blinded investigators scored sections independently; discrepancies were resolved by consensus. For statistics, the per-mouse mean islet area (across sections/islets) was used as a single biological replicate to avoid pseudoreplication.

### Western blot analysis

**Figure 1:**
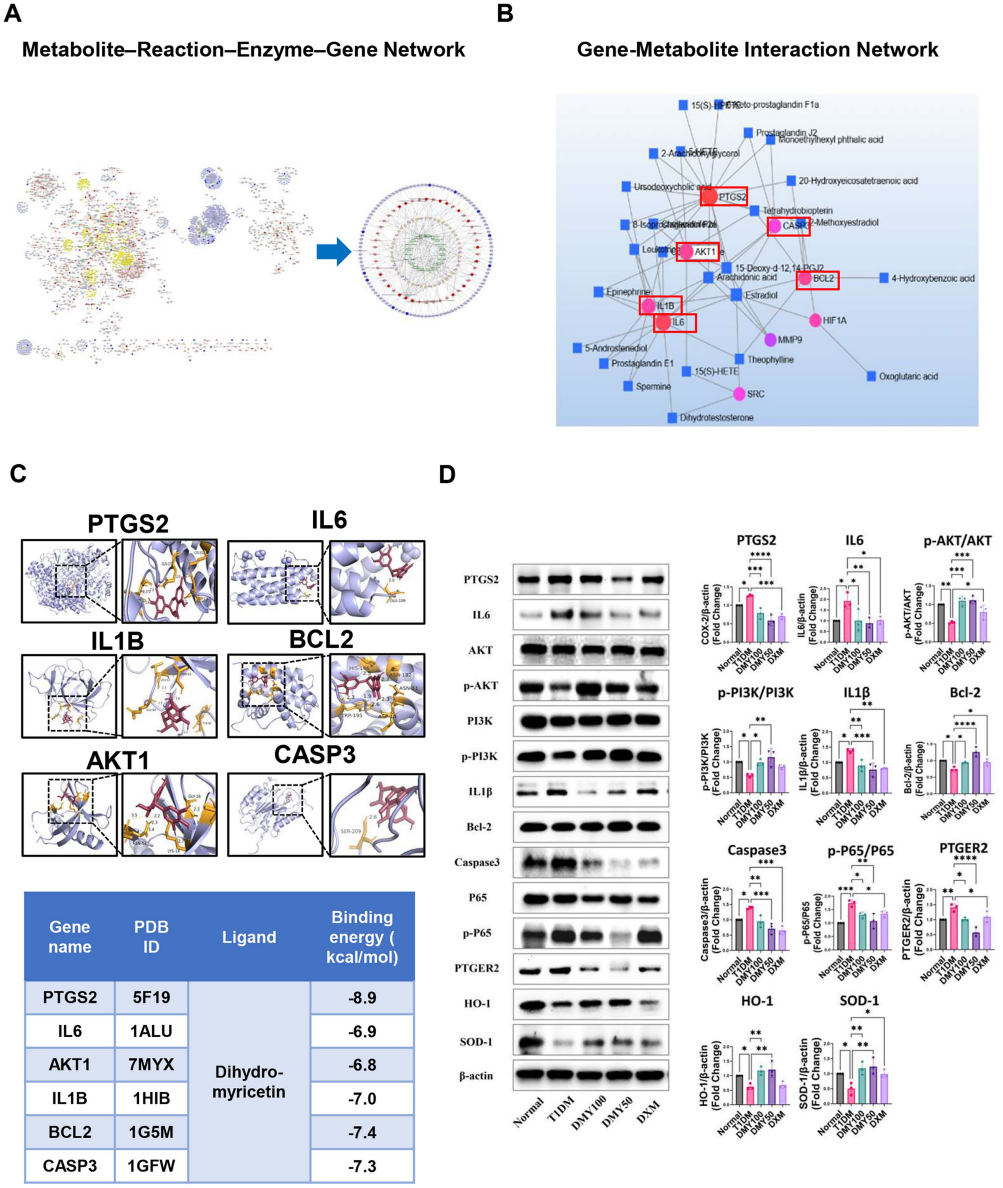
Integrated metabolomics and network pharmacology analysis identifies core therapeutic targets and pathways of DMY in T1DM. **(A)** Metscape-based metabolite–reaction–enzyme–gene (M–R–E–G) network integrating differential metabolites and shared gene targets; arachidonic acid metabolism highlighted as a central module. **(B)** Gene–metabolite interaction network identifying six hub targets (PTGS2, IL6, AKT1, IL1B, BCL2, CASP3) by topological analysis. **(C)** Molecular docking of DMY with hub proteins; representative binding conformations and affinities shown. **(D)** Immunoblot validation in pancreatic tissue. Left: representative blots for PTGS2, IL6, AKT, p-AKT, PI3K, p-PI3K, IL1β, BCL-2, cleaved-caspase-3, P65, p-P65, PTGER2, HO-1, SOD-1, and β-actin. Right: quantification. Phosphorylation is expressed as p-PI3K/PI3K, p-AKT/AKT, and p-P65/P65 ratios (total forms shown on the left); PTGS2, IL6, IL1β, BCL-2, cleaved-caspase-3, PTGER2, HO-1, and SOD-1 are normalized to β-actin. Data are presented as mean ± SD (*n* = 3). Statistical analysis: One-way ANOVA with Tukey’s post hoc test. *p < 0.05, **p < 0.01, ***p < 0.001, ****p < 0.0001 vs. indicated comparison.

**Figure 2:**
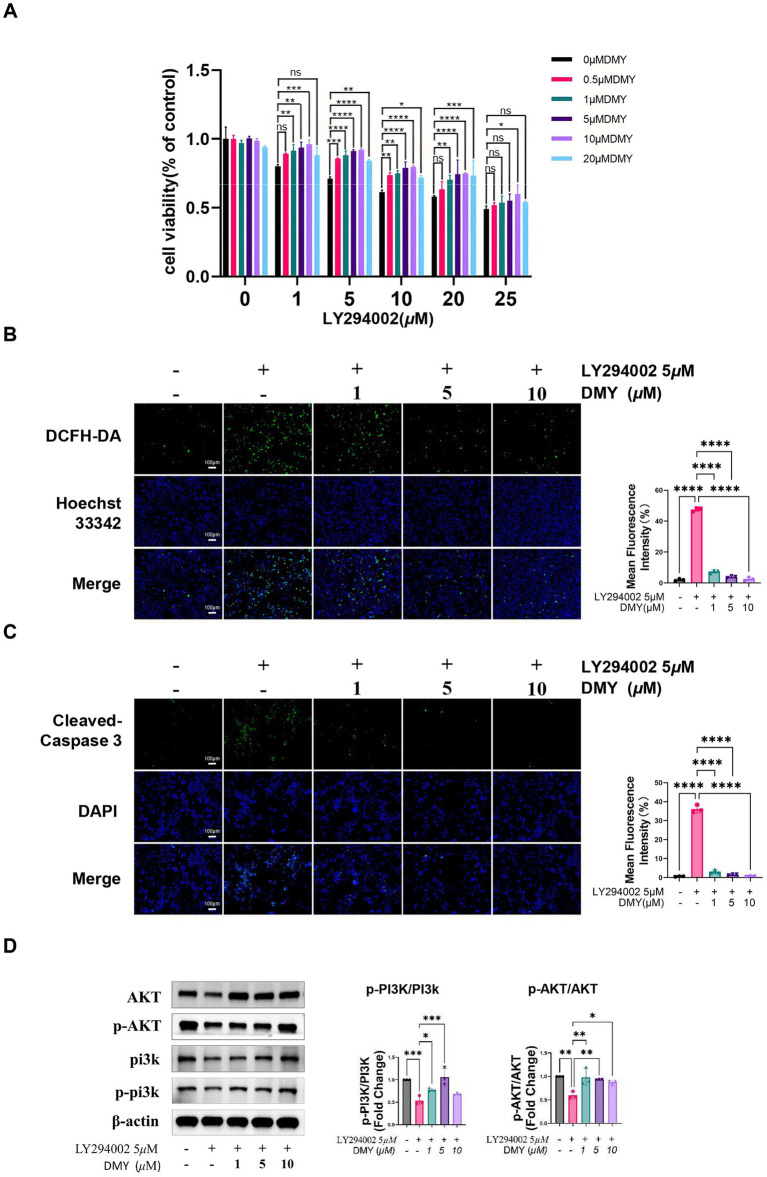
DMY protects INS-1 β-cells from PI3K inhibition–induced oxidative stress and apoptosis via reactivation of the PI3K/AKT pathway. **(A)** Cell viability of INS-1 cells treated with increasing concentrations of LY294002 (0–25 μM) in the presence or absence of DMY (0.5–20 μM), assessed using the CCK-8 assay. **(B)** Intracellular ROS accumulation detected by DCFH-DA fluorescent staining following LY294002 (5 μM) exposure, with or without DMY co-treatment (1, 5, 10 μM). Right panel: quantification of mean fluorescence intensity. **(C)** Immunofluorescence staining for cleaved caspase-3 and DAPI nuclear staining in cells treated as in **(B)**. Right panel: quantification of apoptotic signal intensity. **(D)** Western blot analysis of total and phosphorylated PI3K and AKT in INS-1 cells co-treated with LY294002 and DMY (1, 5, 10 μM). Bar graphs show p-PI3K/PI3K and p-AKT/AKT ratios; totals are shown for reference and β-actin verifies equal loading. Data are presented as mean ± SD (*n* = 3). Statistical analysis was performed using one-way ANOVA with Tukey’s post hoc test. ns, not significant; *p < 0.05, **p < 0.01, ***p < 0.001, ****p < 0.0001 vs. indicated comparison.

For *in vivo* experiments, pancreatic tissue lysates were analyzed (corresponding to Figure 1), whereas for *in vitro* experiments, INS-1 cell lysates were used (corresponding to Figure 2). Tissues were lysed in RIPA buffer with protease/phosphatase inhibitors. Protein concentration was determined by BCA assay. Equal protein amounts were separated by SDS-PAGE, transferred to PVDF membranes, blocked, and probed overnight at 4 °C with primary antibodies. After 1 h incubation with HRP-conjugated secondary antibodies, bands were detected by ECL and quantified with ImageJ v1.54g.

### Plasma metabolomics profiling

Plasma (100 μL) was extracted with cold methanol (1:4, v/v), vortexed, sonicated (4 °C, 1 h), centrifuged (15,000 g, 10 min), dried under N_2_ and reconstituted in acetonitrile: water (1:1, v/v). Samples were randomized and analyzed on a Waters ACQUITY UPLC–Xevo G2-XS QTOF system (BEH C18 2.1 × 50 mm, 1.8 μm) in both ESI + and ESI − modes. LockSpray with leucine enkephalin (*m*/*z* 556.2771/554.2615) provided real-time mass correction. Key settings: capillary 3.0 kV, cone 40 V, desolvation gas 800 L/h (450 °C), source 100 °C. Pooled QC injections every 8–10 runs and blanks monitored stability and carry-over. Raw data were processed in MetaboAnalyst 6.0. After peak filtering (intensity > 100), PCA, OPLS-DA, volcano plot and heatmap analyses were performed. Significant features: fold change > 1.5, *p* < 0.05 (FDR-adjusted). Metabolite IDs were assigned via HMDB and KEGG, and pathway enrichment used MetaboAnalyst.

### Network pharmacology and multi-omics integration

The canonical SMILES of DMY (PubChem CID: 5281605) was retrieved from PubChem and used for target prediction via SwissTargetPrediction (probability > 0), TCMSP, and PharmMapper (version 2017; top 300 pharmacophore models with pKd ≥ 6.0). Predicted protein targets were converted to human gene symbols using UniProt, and cross-referenced against T1DM-related genes collected from GeneCards (relevance score ≥ 1), OMIM, TTD, and DrugBank. After deduplication, 380 unique DMY-related targets were retained.

The intersection with T1DM-associated genes was visualized with Venny 2.1, yielding 167 shared targets. These were imported into STRING (v11.5, confidence > 0.7, *Homo sapiens*) to build a protein–protein interaction (PPI) network, visualized in Cytoscape 3.10.2. Core targets were identified using cytoNCA (topological analysis), MCODE (module clustering), and cytoHubba (multi-algorithm ranking). GO and KEGG enrichment analyses were performed using DAVID v6.8 (*p* < 0.05, Benjamini-adjusted).

For multi-omics integration, differential plasma metabolites (from volcano plot) and shared gene targets were analyzed in MetaboAnalyst 6.0 for KEGG global metabolic network mapping. The Metscape plugin in Cytoscape was used to construct metabolite–reaction–enzyme–gene (M–R–E–G) networks. Hub nodes were defined by degree > 3 and betweenness > 7.21 (29, 30).

### Molecular docking analysis

The 3D structure of dihydromyricetin (DMY; PubChem CID: 5281605) was downloaded in SDF format from PubChem and energy-minimized in Chem3D (MM2 force field), then converted to mol2 format. Crystal structures of hub proteins were retrieved from the RCSB PDB database. All water molecules and heteroatoms were removed, and polar hydrogens and Gasteiger charges were added using AutoDock Tools 1.5.6. Docking was performed using AutoDock Vina (v1.1.2), with grid boxes centered on the active site of each protein, exhaustiveness set to 8. For each target, the pose with the lowest binding energy was selected for analysis. Docking scores (kcal/mol) and binding interactions were visualized in PyMOL 2.5.

### Cell-based validation in INS-1 β-cells

INS-1 cells were cultured in RPMI-1640 supplemented with 10% fetal bovine serum (FBS) and 1% penicillin–streptomycin at 37 °C in a humidified incubator with 5% CO_2_. Cells were seeded in 96-well plates (2 × 10^4^ cells/well) 24 h before treatment. To model cytoprotection under ongoing kinase inhibition (rescue paradigm), INS-1 cells were exposed to LY294002 (5 μM, 24 h) with or without co-incubation of DMY (1, 5, or 10 μM). For dose–response viability assays, LY294002 (0–25 μM) and DMY (0.5–20 μM) were tested for 24 h; for ROS, immunofluorescence, and Western blot assays, LY294002 was used at 5 μM with DMY at 1, 5, or 10 μM for 24 h. Cell viability was measured using the CCK-8 assay (GLPBIO). Intracellular ROS was detected by DCFH-DA staining (10 μM; Beyotime) and imaged by fluorescence microscopy. Apoptosis was assessed by immunofluorescence staining of cleaved caspase-3 (Affinity) with DAPI counterstaining (Pusiteng). Phosphorylated PI3K (Tyr458) and AKT (Ser473) and their total forms were analyzed by Western blot. All experiments were performed in biological triplicates unless otherwise stated.

### Statistical analysis

Statistical analysis was performed using GraphPad Prism version 9.5.0 (GraphPad Software, USA). All data are presented as mean ± standard deviation (SD). For comparisons among multiple groups, one-way analysis of variance (ANOVA) followed by Tukey’s *post hoc* test was used. For comparisons between two groups, unpaired Student’s *t*-test was applied. A *p*-value < 0.05 was considered statistically significant. Statistical significance is indicated as follows: *p < 0.05, **p < 0.01, ***p < 0.001, ****p < 0.0001.

## Results

### DMY alleviates diabetic symptoms and preserves β-cell function

**Figure 3:**
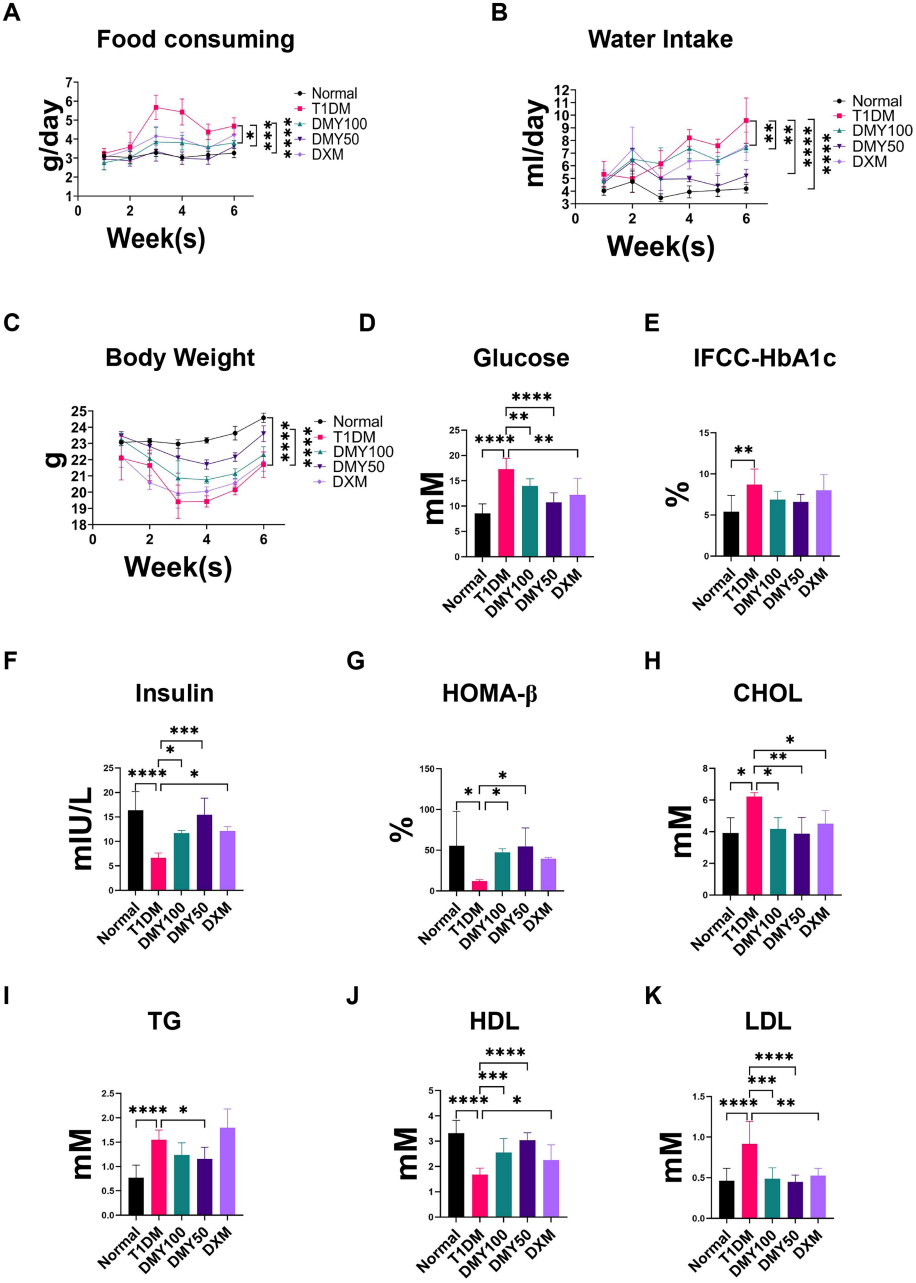
Dihydromyricetin (DMY) alleviates diabetic symptoms and improves β-cell function in streptozotocin (STZ)-induced T1DM mice. **(A–C)** Food consumption, water intake, and body weight were recorded weekly. **(D)** Fasting blood glucose (FBG) levels. **(E)** Glycated hemoglobin (IFCC-HbA1c) percentage. **(F)** Plasma insulin levels. **(G)** HOMA-β index calculated to estimate β-cell functional capacity. **(H–K)** Serum lipid profiles including total cholesterol (TC), triglycerides (TG), high-density lipoprotein cholesterol (HDL-C), and low-density lipoprotein cholesterol (LDL-C). Data are presented as mean ± SD (*n* = 10–12 per group). **(A–C)** Weekly measures from the pre-treatment week through the post-STZ period. **(D–K)** Terminal fasting measurements collected at the study endpoint. Statistical analysis was performed using one-way ANOVA followed by Tukey’s *post hoc* test. *p < 0.05, **p < 0.01, ***p < 0.001, ****p < 0.0001 vs. T1DM group unless otherwise indicated.

After STZ induction, T1DM mice displayed significant weight loss, polyphagia, polydipsia, and elevated fasting blood glucose (FBG; *p* < 0.0001). DMY, especially at 50 mg/kg, markedly improved body weight and normalized food and water intake (Figures 3A–C). FBG levels were significantly reduced by DMY (Figure 3D), and although HbA1c trended downward, it did not reach significance (Figure 3E). Plasma insulin levels, which had fallen sharply in T1DM mice (*p* < 0.01), were restored by DMY50 (*p* < 0.0001) and DMY100 (*p* < 0.01; Figure 3F). HOMA-β index, an estimate of β-cell function, increased in both treatment groups (*p* < 0.01; Figure 3G). DMY also corrected STZ-induced dyslipidemia. T1DM mice exhibited higher total cholesterol (TC, *p* < 0.05), triglycerides (TG, *p* < 0.0001), and LDL-C (*p* < 0.0001), alongside lower HDL-C (*p* < 0.0001). DMY50 significantly decreased TC, TG and LDL-C, while markedly increasing HDL-C (Figures 3H–K). During the 7-day pre-STZ period, body weight, food intake, and water intake did not differ among groups; group differences emerged after STZ induction, with DMY attenuating these abnormalities.

### DMY preserves islet morphology and attenuates pancreatic injury

**Figure 4:**
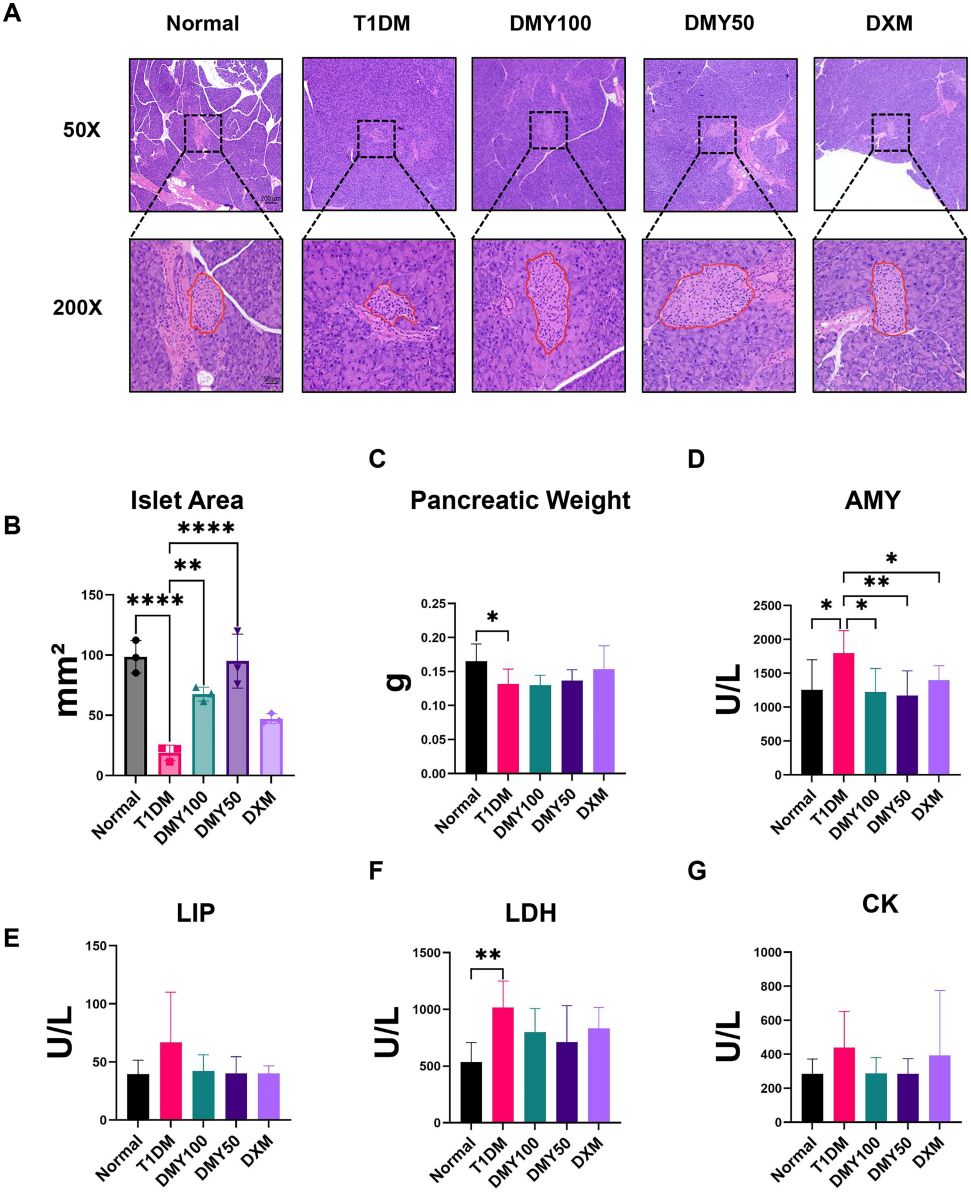
Dihydromyricetin (DMY) improves pancreatic islet morphology and alleviates STZ-induced pancreatic injury in T1DM mice. **(A)** Representative H&E-stained pancreatic sections from each group (50 × and 200 × magnifications). Islet boundaries are outlined in red at 200×. Representative images were randomly selected from the pool of quantified sections under blinded conditions. **(B)** Quantification of per-mouse mean islet area (mm^2^) calculated across three equidistant sections per pancreas. **(C)** Pancreatic weight at study endpoint. **(D–G)** Plasma biochemical markers of pancreatic injury, including amylase (AMY), lipase (LIP), lactate dehydrogenase (LDH), and creatine kinase (CK). Data are presented as mean ± SD (*n* = 6 per group for histology, *n* = 8–10 per group for biochemical assays). Statistical analysis was performed using one-way ANOVA followed by Tukey’s post hoc test. *p < 0.05, **p < 0.01, ***p < 0.001, ****p < 0.0001 vs. T1DM group unless otherwise indicated.

H&E staining revealed shrunken, disorganized islets in T1DM mice, whereas DMY50 restored islet architecture most effectively (Figure 4A). Quantification confirmed a drastic islet-area reduction in T1DM mice (*p* < 0.0001), which was significantly reversed by DMY50 (*p* < 0.0001) and DMY100 (*p* < 0.05; Figure 4B). Pancreatic weight declined in T1DM mice (*p* < 0.05; Figure 4C) and was unaffected by treatment. Plasma amylase (AMY) rose in T1DM mice (*p* < 0.05) and was normalized by DMY50 (*p* < 0.01) and DMY100 (*p* < 0.05; Figure 4D). Lipase (LIP) and creatine kinase (CK) levels remained stable across groups. Lactate dehydrogenase (LDH) increased in T1DM mice (*p* < 0.01) but was not significantly reduced by DMY and DXM treatment (Figures 4E–G). Having demonstrated DMY’s systemic benefits on glycemia, lipids, and islet integrity, we next investigated its impact on the plasma metabolome.

### DMY50 reprograms the plasma metabolome in T1DM mice

**Figure 5:**
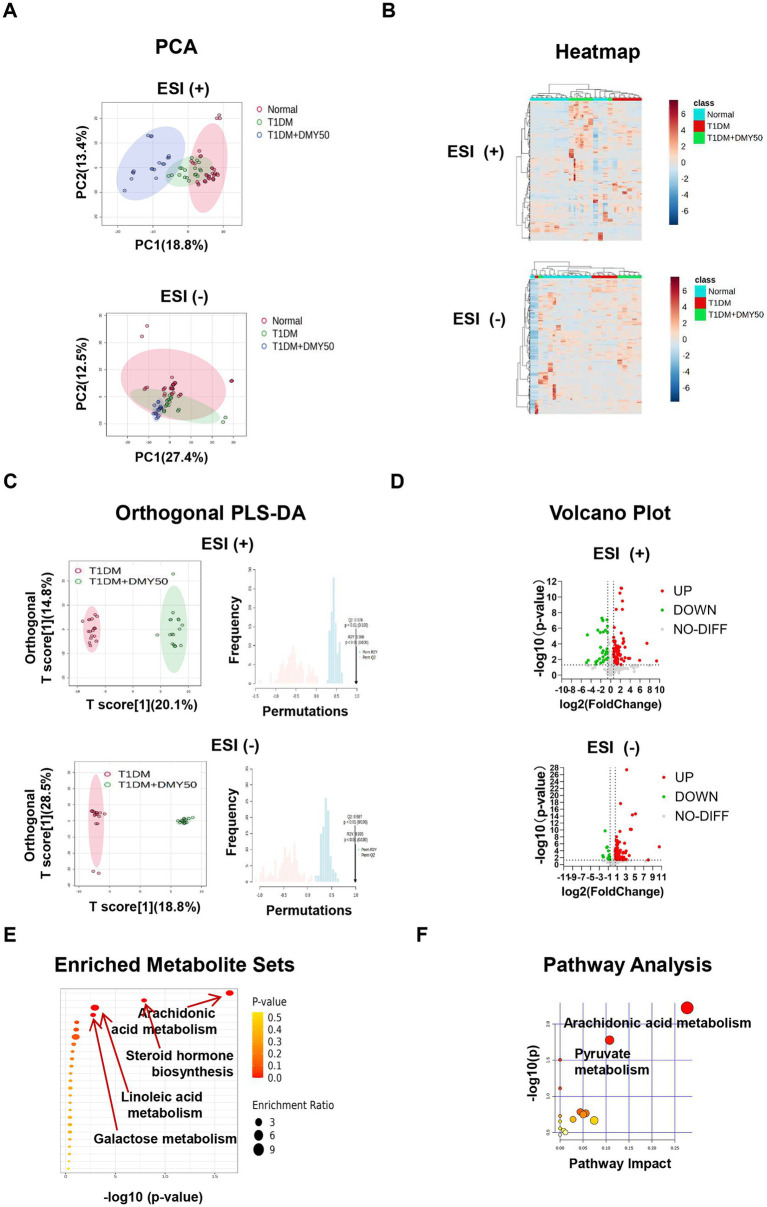
Untargeted plasma metabolomics of Normal, T1DM and DMY50-treated mice. **(A)** PCA in positive and negative ion modes. **(B)** Heatmap of top differential metabolites. **(C)** OPLS-DA with permutation validation. **(D)** Volcano plots of significant metabolites. **(E)** Pathway enrichment bubble plot. **(F)** KEGG pathway analysis of selected metabolites. Data processed in MetaboAnalyst 6.0 (fold change and *p*-value criteria set in Methods).

In ESI-positive mode, 3,964 ions (285 metabolites) and in ESI-negative mode, 3,747 ions (286 metabolites) passed quality filters (Supplementary Tables S1, S2). PCA showed clear separation of Normal, T1DM, and DMY50 groups (Figure 5A), and heatmap clustering placed DMY50 closer to Normal (Figure 5B). OPLS-DA distinguished T1DM from DMY50 with permutation-validated robustness (Figure 5C). Volcano analysis identified 189 differential metabolites (*p* < 0.05, FC > 1.5), including 69 up/30 down in ESI + and 76 up/14 down in ESI − (Figure 5D). Enrichment highlighted arachidonic acid, steroid hormone, linoleic acid and galactose metabolism (Figure 5E). Twenty-eight key metabolites were selected for a focused KEGG network, underscoring arachidonic acid and pyruvate pathways (Figure 5F; Table 1). To link these metabolic alterations to molecular targets of DMY, we conducted network pharmacology analysis.

**Table 1:** Differential endogenous metabolites between T1DM and DMY50 groups.

Metabolite	Formula	Adducts	*m*/*z*	Retention time (min)	HMDB ID	KEGG ID	AUC	*p*-value	Fold change (log_2_)
Estriol	C18H24O3	M + H	289.1813	0.96	HMDB0000153	C05141	1.00	4.61E−12	−2.3967
9-Hydroxyandrost-4-ene-3,17-dione	C19H26O3	M + H	303.1927	1.00	HMDB0003955	C05290	1.00	5.46E−14	−2.2318
15-Deoxy-d-12,14-PGJ2	C20H28O3	M + H	317.2091	1.05	HMDB0005079	C14717	1.00	2.55E−14	−2.1212
Arachidonic acid	C20H32O2	M + ACN + H	346.2744	0.56	HMDB0001043	C00219	1.00	4.76E−07	4.7691
13-OxoODE	C18H30O3	M + H	295.2264	1.07	HMDB0004668	C14765	0.99	2.65E−07	−2.1491
(N-acetylneuraminosyl(a2-6)lactosamine)	C25H42N2O19	M + H	675.2487	3.29	HMDB0001081	C04886	0.99	2.06E−07	2.1379
12S-HHT	C17H28O3	M + H	281.2106	1.00	HMDB0012535	C20388	0.99	7.71E−11	−2.3848
Malic acid	C4H6O5	M + 2H	68.0177	0.04	HMDB0000156	C00149	0.97	2.62E−09	0.7396
Tetrahydrobiopterin	C9H15N5O3	M + H	242.1264	0.87	HMDB0000027	C00272	0.97	1.57E−06	−2.5801
17alpha-Estradiol	C18H24O2	M + H	273.1881	0.97	HMDB0000429	C02537	0.97	8.76E−08	2.6127
Aquacobalamin	C72H105CoN16O20PS	M + 2H	818.8193	0.61	HMDB0003458	C00992	0.95	2.14E−08	0.6588
4-Pyridoxic acid	C8H9NO4	M + H	184.0589	2.91	HMDB0000017	C00847	0.95	3.29E−05	−1.8373
14,15-DiHETrE	C20H34O4	M + H	339.2546	1.17	HMDB0002265	C14775	0.95	3.79E−05	−2.9407
Spermine	C10H26N4	M + H	203.2209	0.71	HMDB0001256	C00750	0.93	1.90E−07	0.6335
Heparan sulfate	C14H25NO21S3	M + H	640.0235	0.74	HMDB0000693	C00925	0.93	1.58E−07	1.5276
Lignans	C22H22O8	M + H	415.1345	1.44	HMDB0031452	C10871	0.92	2.33E−05	−1.4383
Epinephrine	C9H13NO3	M + H	184.0971	0.80	HMDB0000068	C00788	0.91	1.01E−06	−0.6195
15-HETE	C20H32O3	M + ACN + H	362.265	1.53	HMDB0003876	C04742	0.90	9.82E−05	−1.0362
Acetic acid	C2H4O2	M + H	61.0286	2.80	HMDB0000042	C00033	0.90	9.97E−06	−2.5062
Hydroxyphenyllactic acid	C9H10O4	M + H	183.0669	0.91	HMDB0000755	C03672	0.90	3.72E−09	1.5923
p-Cresol	C7H8O	M − H	107.0511	0.92	HMDB0001858	C01468	1.00	1.45E−12	−4.1928
Ursodeoxycholic acid	C24H40O4	M − H	391.2848	1.72	HMDB0000946	C07880	0.95	4.38E−05	−2.4185
12,13-DHOME	C18H34O4	M − H	313.2381	1.22	HMDB0004705	C14829	0.91	3.48E−06	−0.67776
Cytidine	C9H13N3O5	M − H	242.0809	0.80	HMDB0000089	C00475	0.90	5.98E−06	−0.65685
9,12,13-TriHOME	C18H34O5	M − H	329.2328	0.94	HMDB0004708	C14833	0.90	1.17E−04	−1.4531
Prostaglandin J2	C20H30O4	M − H	333.2064	1.31	HMDB0002710	C05957	0.90	6.72E−06	−1.0713
Prostaglandin E1	C20H34O5	M − H	353.2313	1.02	HMDB0001442	C04741	0.90	7.90E−05	−2.2758
Chenodeoxycholic acid	C24H40O4	M − H	391.2853	2.62	HMDB0000518	C02528	0.90	7.53E−06	−1.7355

This table lists the top 28 endogenous metabolites significantly altered by DMY50 treatment in T1DM mice, identified via untargeted UPLC-QTOF-MS analysis. Each metabolite is annotated with chemical formula, ion adduct type, *m*/*z* value, retention time, HMDB ID, KEGG ID, area under the curve (AUC), statistical *p*-value, and log_2_ fold change (T1DM vs. DMY50). Positive log_2_FC values indicate upregulation in T1DM, and negative values reflect upregulation by DMY50.

### Network pharmacology identifies PI3K/AKT Signaling as a Core therapeutic target of DMY

**Figure 6:**
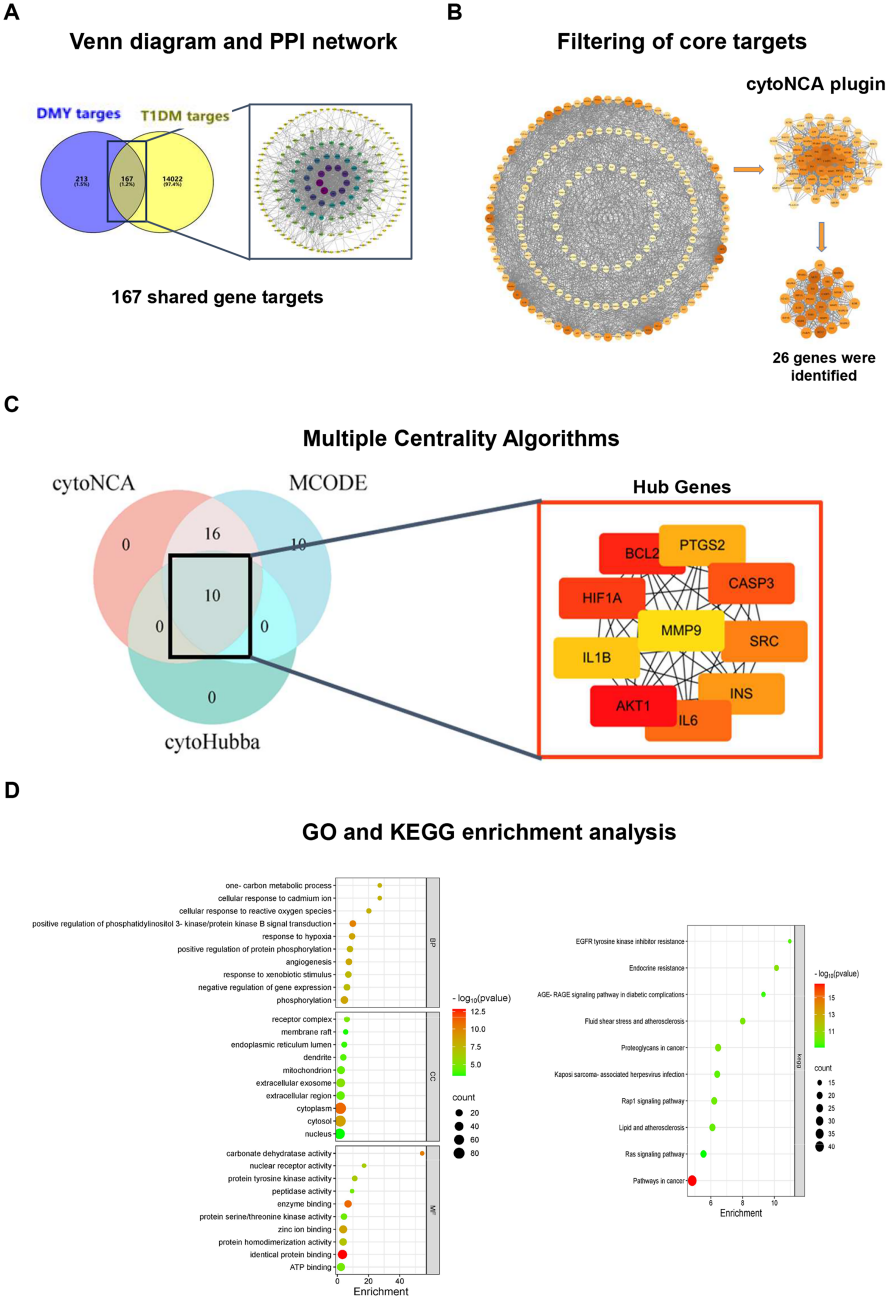
Network pharmacology analysis identifies hub genes and enriched pathways related to the antidiabetic effects of DMY. **(A)** Venn diagram showing the overlap between predicted DMY targets (*n* = 380) and T1DM-related genes (*n* = 14,022), yielding 167 shared targets; STRING-based PPI network visualized in Cytoscape. **(B)** Core gene selection by cytoNCA (six topological parameters; two-step median filtering, *n* = 26). **(C)** Hub gene identification by integrating cytoNCA, MCODE (degree cutoff = 2, node score cutoff = 0.2, k-core = 2), and cytoHubba (top 10 by MCC). Overlap yielded 10 hub genes: AKT1, BCL2, SRC, CASP3, INS, IL6, HIF1A, PTGS2, MMP9, IL1B. Node color intensity reflects degree centrality; node size indicates network connectivity. **(D)** GO and KEGG enrichment analysis (DAVID v6.8, Benjamini-adjusted *p* < 0.05). Left: Top 10 terms for biological process (BP), cellular component (CC), and molecular function (MF). Right: Enriched KEGG pathways including PI3K-AKT, AGE-RAGE, and HIF-1 signaling.

Network pharmacology analysis identified 380 candidate targets of DMY, and intersection with T1DM-related genes yielded 167 shared targets (Figure 6A). The STRING-based PPI network consisted of 155 nodes and 1,480 edges, with node color intensity indicating degree centrality. Using cytoNCA, two-step median filtering selected 26 core genes (Figure 6B). Additional analyses using MCODE and cytoHubba identified key modules and ranked hub genes; the overlap among methods yielded 10 hub genes: AKT1, BCL2, SRC, CASP3, INS, IL6, HIF1A, PTGS2, MMP9, and IL1B (Figure 6C). GO enrichment revealed significant associations with PI3K-AKT signaling, oxidative stress response, hypoxia, angiogenesis, and gene expression regulation. KEGG pathway analysis further identified enrichment in PI3K-AKT, HIF-1, lipid metabolism, AGE-RAGE, atherosclerosis, and endocrine resistance pathways (Figure 6D). These findings highlight PI3K/AKT signaling as a central axis potentially mediating the therapeutic effects of DMY in T1DM. Building on these core targets, we then performed an integrated metabolomics–network pharmacology analysis to pinpoint key therapeutic hubs. Although PI3K was not ranked among the top hub genes by centrality, pathway enrichment consistently highlighted the PI3K/AKT axis (with AKT1 as a hub); therefore, PI3K was included as an upstream node for causal validation.

### Integrated metabolomics and network pharmacology reveal six core targets of DMY in T1DM

To link metabolic changes with gene-level regulation, KEGG global metabolic network analysis (MetaboAnalyst 6.0) was performed by integrating 189 differential plasma metabolites and 167 shared gene targets of DMY and T1DM. This analysis identified nine significantly enriched pathways (*p* < 0.05), with arachidonic acid metabolism, steroid hormone biosynthesis, and linoleic acid metabolism among the most prominent (Table 2). Using Cytoscape 3.10.2 and the Metscape plugin, a metabolite–reaction–enzyme–gene (M–R–E–G) network was constructed, highlighting arachidonic acid metabolism as a central node (Figure 1A). Parallel gene–metabolite interaction analysis identified six hub genes—PTGS2, IL6, AKT1, IL1B, BCL2, and CASP3—based on topological criteria (degree > 3, betweenness > 7.21; Figure 1B, Table 3). These targets represent critical intersection points between inflammatory and metabolic pathways.

**Table 2:** KEGG pathway enrichment of integrated differential metabolites and gene targets.

Name	Hits	*p*-value
Arachidonic acid metabolism	28	5.28E−27
Steroid hormone biosynthesis	22	3.39E−15
Linoleic acid metabolism	6	0.0000054
Galactose metabolism	8	0.00101
Caffeine metabolism	3	0.00892
Glycosaminoglycan biosynthesis—keratan sulfate	2	0.0128
Lipoic acid metabolism	4	0.0291
Primary bile acid biosynthesis	4	0.0307
Tyrosine metabolism	6	0.0375

This table lists the top 9 enriched KEGG pathways identified by integrating 189 differential plasma metabolites with 167 DMY–T1DM shared gene targets. Enrichment was performed using MetaboAnalyst 6.0 (pathway topology analysis) and DAVID v6.8. Pathways were ranked by *p*-value significance (cutoff *p* < 0.05) and number of involved components (“Hits”).

**Table 3:** Hub genes in the gene–metabolite interaction network.

Id	Label	Degree	Betweenness
5,743	PTGS2	17	236.27
3,569	IL6	13	145.89
207	AKT1	8	56.18
3,553	IL1B	8	42.37
596	BCL2	6	53.52
836	CASP3	5	15.86

Hub genes were identified by topological analysis of the gene–metabolite interaction network using degree and betweenness centrality thresholds (degree > 3, betweenness > 7.21). The table lists each hub’s gene/protein name, unique identifier, connectivity (degree), and betweenness score. These genes represent key regulatory nodes linking metabolic changes to transcriptional targets in DMY-treated T1DM mice.

Molecular docking supported direct interactions between DMY and the six predicted targets (PTGS2, IL6, AKT1, IL1B, BCL2, CASP3; Figure 1C). In pancreatic tissue, immunoblotting (Figure 1D) showed that DMY50 lowered pro-inflammatory mediators (PTGS2, IL6, IL1B) and the pro-apoptotic marker cleaved-caspase-3, while increasing the anti-apoptotic protein BCL2; total AKT levels were maintained. Pathway activity was quantified as phospho/total ratios from the same membranes. DMY50 significantly increased the p-PI3K/PI3K and p-AKT/AKT ratios and decreased the p-P65/P65 ratio, indicating activation of PI3K/AKT and attenuation of NF-κB signaling. In parallel, PTGER2 was reduced, whereas the antioxidant enzymes HO-1 and SOD1 were upregulated (β-actin-normalized), consistent with anti-inflammatory and cytoprotective effects (Figure 1D). Altogether, these integrated multi-omics results indicate that PI3K/AKT–eicosanoid signaling is a central therapeutic axis of DMY action in T1DM. The six hub genes function as key regulators coordinating lipid-mediated inflammation, oxidative stress, apoptosis, and β-cell survival. To validate the causal role of the identified PI3K/AKT–eicosanoid axis, we turned to an *in vitro* INS-1 β-cell model.

### DMY attenuates oxidative stress and apoptosis in INS-1 β-cells via PI3K/AKT pathway activation

To determine whether DMY directly protects β-cells via the PI3K/AKT pathway, INS-1 cells were exposed to the PI3K inhibitor LY294002. LY294002 significantly reduced cell viability in a dose-dependent manner, while co-treatment with DMY (1–10 μM) markedly restored cell survival at 5 μM LY294002, with the strongest effect observed at higher concentrations (Figure 2A).

Fluorescent staining showed that LY294002 led to a pronounced increase in intracellular ROS, which was significantly reduced by DMY in a concentration-dependent fashion (Figure 2B, *p* < 0.0001). Immunofluorescence analysis indicated that PI3K inhibition enhanced caspase-3 activation and apoptosis, whereas DMY co-treatment substantially decreased cleaved caspase-3 levels (Figure 2C).

Western blot analysis further demonstrated that LY294002 suppressed p-PI3K/PI3K and p-AKT/AKT, whereas co-treatment with DMY restored both ratios (Figure 2D). Together, these findings demonstrate that DMY protects INS-1 β-cells against PI3K inhibition–induced oxidative stress and apoptosis by maintaining PI3K/AKT signaling activity.

In summary, these results demonstrate DMY’s protective effects at systemic, metabolic, and molecular levels, which we next examine in the context of existing literature.”

## Discussion

This study provides systems-level evidence that dihydromyricetin (DMY) exerts multifaceted protective effects in T1DM. DMY lowered fasting glucose, improved lipid profiles, and enhanced β-cell function, as indicated by increased insulin secretion and HOMA-β. Histological and biochemical analyses confirmed islet preservation and reduced pancreatic injury. Untargeted metabolomics showed that DMY modulated key metabolic pathways, including arachidonic acid, steroid hormone, and linoleic acid metabolism. These systemic effects align with prior reports of DMY’s metabolic benefits and extend its role to autoimmune β-cell protection.

These effects are consistent with previous findings that DMY improves insulin sensitivity and lipid profiles in metabolic models (10, 13). The observed reduction in triglycerides and LDL-C is clinically relevant, as lipid accumulation promotes islet inflammation and β-cell loss in both T1DM and T2DM (14). Preservation of islet area and lower serum amylase suggest additional protection of pancreatic integrity (15). Given the pleiotropic metabolic effects of glucocorticoids, DXM was used only as a pharmacological comparator for anti-inflammatory signaling (e.g., NF-κB attenuation) and not as a treatment benchmark; primary efficacy inferences relied on comparisons among Normal, T1DM, and DMY groups.

DMY50 treatment significantly altered the metabolism of certain fatty acids (notably arachidonic acid and linoleic acid). Eicosanoids derived from COX-2 (PTGS2) and 12-LOX contribute to β-cell inflammation and oxidative stress (16, 17), yet regulated production is essential for insulin secretion (18). DMY may help restore balance to the eicosanoid signaling pathway—reducing harmful ROS generation while still supporting necessary lipid signaling. Enrichment of estriol and tetrahydrobiopterin suggests further antioxidant support via steroid hormone biosynthesis. To elucidate how these pathway shifts translate into molecular regulation, we integrated network pharmacology to identify DMY’s core targets.

Network pharmacology identified six core targets—PTGS2, IL6, AKT1, IL1B, BCL2, and CASP3—linking metabolic and gene-level effects. AKT1 promotes β-cell survival and insulin production; BCL2 inhibits mitochondrial apoptosis. In contrast, IL-1β (IL1B) and COX-2 drive ROS and caspase-3 activation (19, 20). DMY upregulated AKT1/BCL2 and downregulated PTGS2/IL1B, shifting the molecular profile toward cell survival. In INS-1 cells, DMY reversed ROS accumulation and caspase-3 cleavage caused by LY294002, a selective class I PI3K inhibitor (21). Restoration of p-PI3K and p-AKT confirms the necessity of PI3K/AKT activation in DMY’s cytoprotective effects. Together, these findings connect metabolic reprogramming, hub-gene modulation, and inhibitor-based validation into a coherent mechanism: DMY protects β-cells by activating PI3K/AKT and suppressing eicosanoid-driven inflammation.

In INS-1 cells, LY294002 produced a modest reduction in total AKT. This phenomenon has been described during apoptosis, where activated caspase-3 cleaves AKT and weakens the full-length band (22), and also when LY294002 suppresses mTORC1, which reduces S6K/4E-BP1 output and global protein synthesis, lowering steady-state AKT during 24-h exposure (23). DMY co-treatment decreased cleaved caspase-3 and restored the p-AKT/AKT ratio, indicating that the apparent fall in total AKT reflects stress-related effects rather than a primary down-regulation of AKT. In view of these context-dependent effects and to test cytoprotection under active PI3K blockade, our *in vitro* assay used a rescue paradigm (co-incubation with LY294002), whereas the *in vivo* schedule modeled prophylaxis (pre- and co-treatment with DMY). We acknowledge that a pre-treatment design in INS-1 cells would better parallel the in vivo schedule and will be addressed in future studies. Although flavonoids are widely studied in T2DM, most work has focused on quercetin, kaempferol, or multi-herbal formulas (24). DMY has shown benefits in hepatic steatosis and diabetic cardiomyopathy (13, 25), but studies on autoimmune β-cell loss are lacking. This study makes three key contributions: (1) demonstration of direct β-cell protection in a canonical T1DM model; (2) application of untargeted metabolomics and network pharmacology to dissect how DMY regulates lipid–inflammation–apoptosis pathways—an approach rarely used for single flavonoids (26, 27); (3) mechanistic validation with LY294002, linking in silico prediction to functional proof. To our knowledge, no previous study has combined these strategies to position PI3K/AKT as a core effector of DMY action in T1DM.

While these approaches support our findings, several limitations remain to be addressed. The STZ model reflects acute oxidative injury rather than the chronic autoimmune progression seen in human T1DM (28). Validation in NOD mice or human islets is needed. Only male mice were studied; sex-specific effects remain unknown. Long-term efficacy, pharmacokinetics, and safety also require investigation. Finally, metabolomics was limited to plasma; tissue-specific or single-cell profiling may yield more mechanistic insight. Finally, we did not measure Bax and therefore could not calculate the Bax/BCL-2 ratio; future studies will incorporate this apoptosis index. Nevertheless, our results provide a strong foundation for future validation and translational development.

## Conclusion

Dihydromyricetin (DMY) markedly reduces hyperglycemia, preserves islet architecture, and restores metabolic homeostasis in a T1DM mouse model. Integrated metabolomic and network pharmacology analyses pinpointed key pathways—arachidonic acid metabolism, steroid hormone biosynthesis, linoleic acid metabolism, and PI3K/AKT signaling—through which DMY exerts its effects. Functional validation demonstrated that DMY activates PI3K/AKT, attenuates oxidative stress, and prevents β-cell apoptosis. By identifying a readily available dietary flavonoid capable of safeguarding β-cells, this study contributes to the search for safe adjunct therapies in T1DM and offers a scalable framework for future nutraceutical development.

## Data Availability

Publicly available datasets were analyzed in this study. This data can be found here: https://pubchem.ncbi.nlm.nih.gov, CID 5281605. Further inquiries can be directed to the corresponding author.

## Glossary

DMY: Dihydromyricetin
T1DM: Type 1 diabetes mellitus
STZ: Streptozotocin
FBG: Fasting Blood Glucose
HbA1c: Glycated Hemoglobin
HDL-C: High-Density Lipoprotein Cholesterol
LDL-C: Low-Density Lipoprotein Cholesterol
HOMA-β: Homeostasis Model Assessment of β-cell Function
ROS: Reactive Oxygen Species
PCA: Principal Component Analysis
OPLS-DA: Orthogonal Partial Least Squares Discriminant Analysis
PPI: Protein–Protein Interaction
M–R–E–G: Metabolite–Reaction–Enzyme–Gene
KEGG: Kyoto Encyclopedia of Genes and Genomes
GO: Gene Ontology
PTGS2: Prostaglandin-Endoperoxide Synthase 2 (COX-2)
IL6: Interleukin 6
AKT1: RAC-alpha Serine/Threonine-Protein Kinase
IL1B: Interleukin 1 Beta
BCL2: B-cell Lymphoma 2
CASP3: Caspase 3
INS-1: Rat Insulinoma-1 Cell Line
