# Supplementary material for: Dihydromyricetin preserves β-cell function in type 1 diabetes via PI3K/AKT-mediated metabolic reprogramming
Source: Front Nutr. 2025 Oct 2;12:1682308. doi: 10.3389/fnut.2025.1682308 (PMC12527859; doi:10.3389/fnut.2025.1682308)

Mouse pancreatic tissue PTGS2 50-68kDa

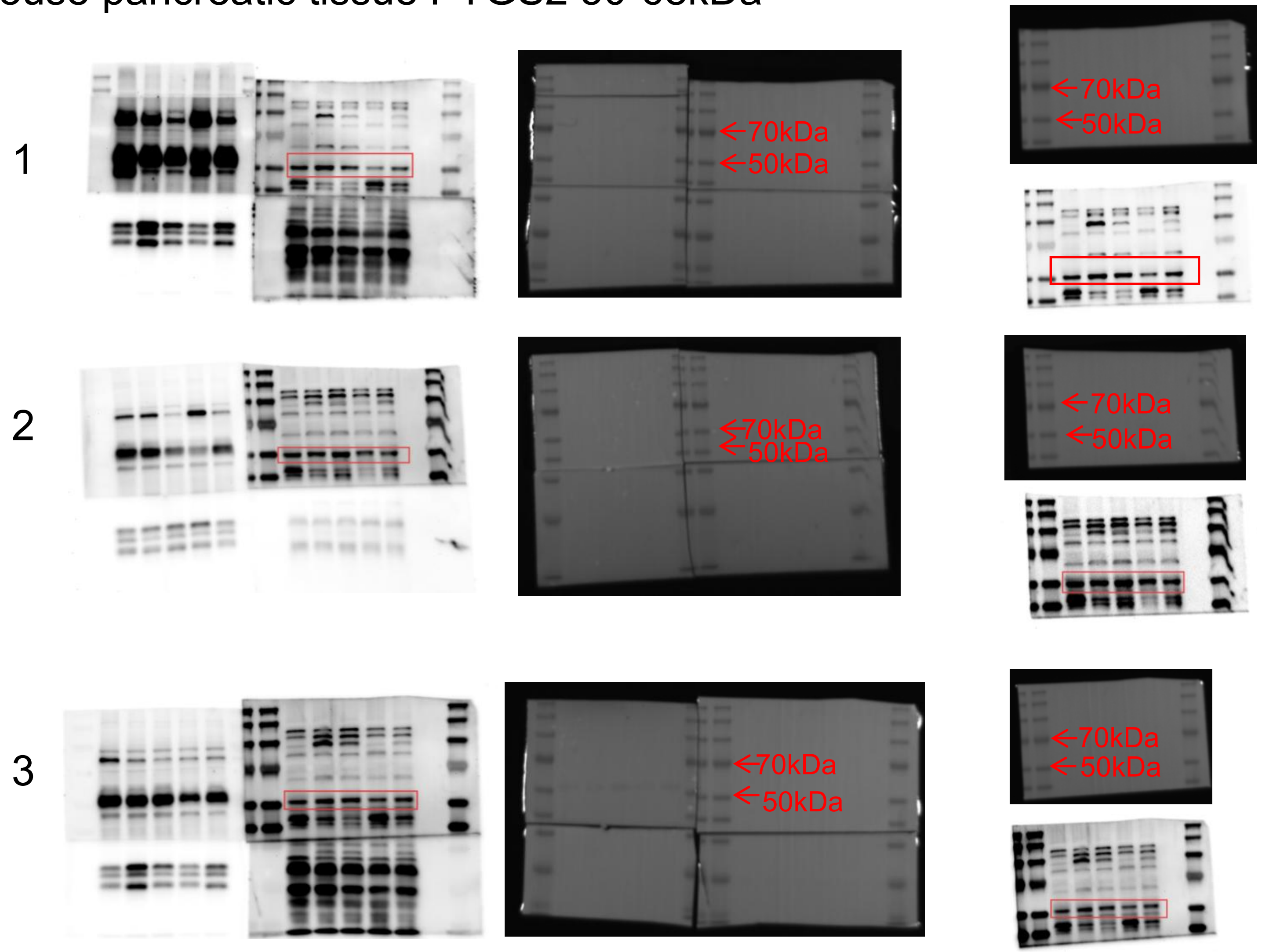

Mouse pancreatic tissue IL6 24kDa

1

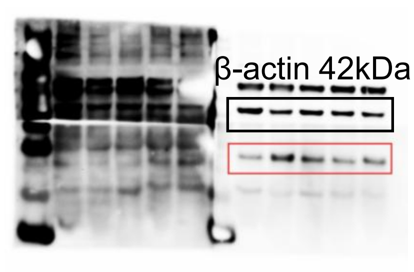

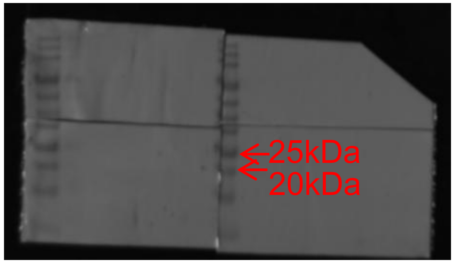

2

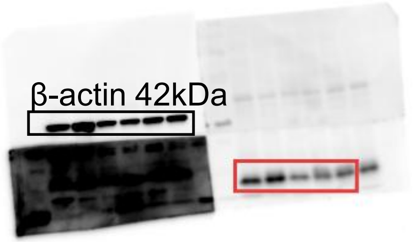

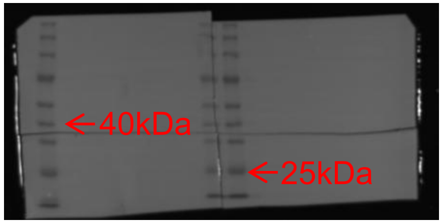

3

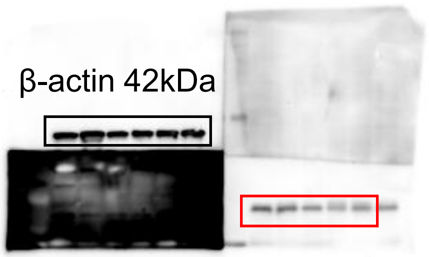

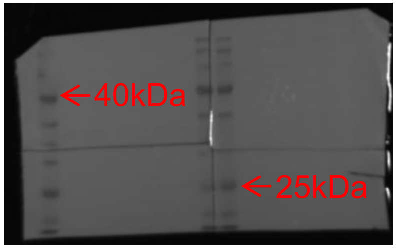

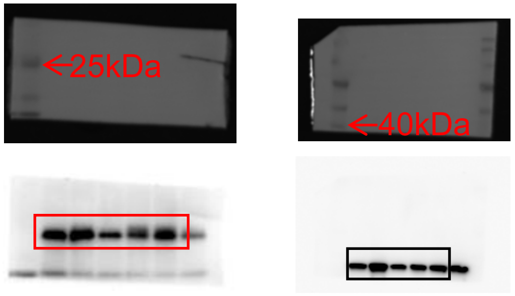

Mouse pancreatic tissue AKT 56kDa

1

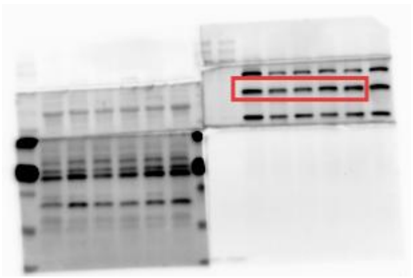

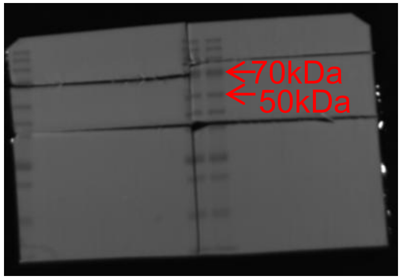

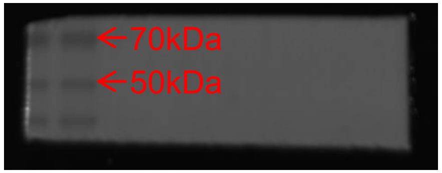

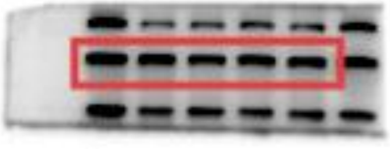

2

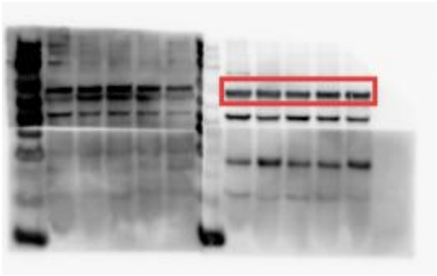

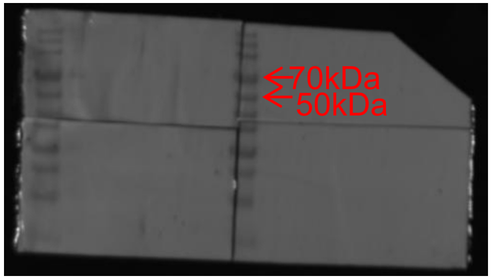

3

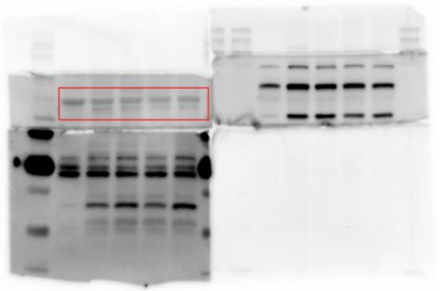

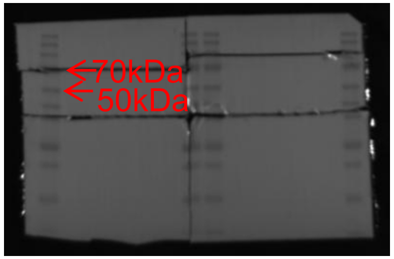

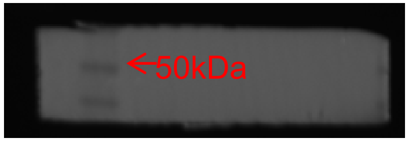

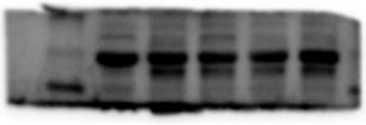

Mouse pancreatic tissue p-AKT 56kDa

1

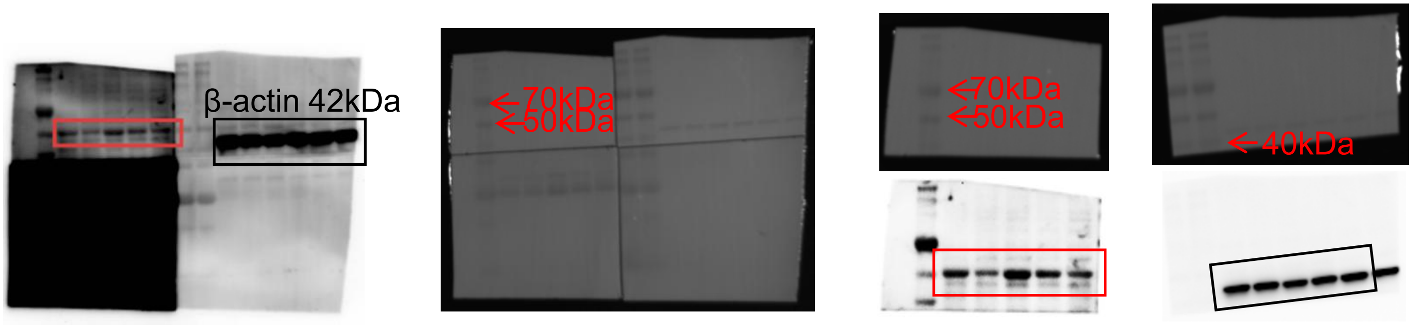

2

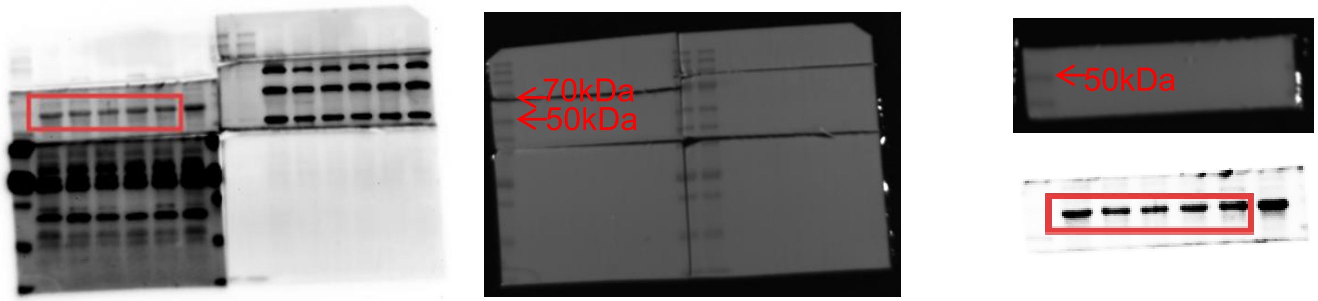

3

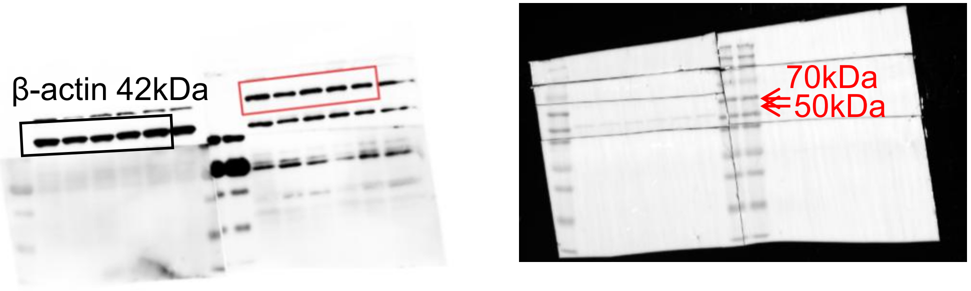

Mouse pancreatic tissue PI3K 55kDa

1

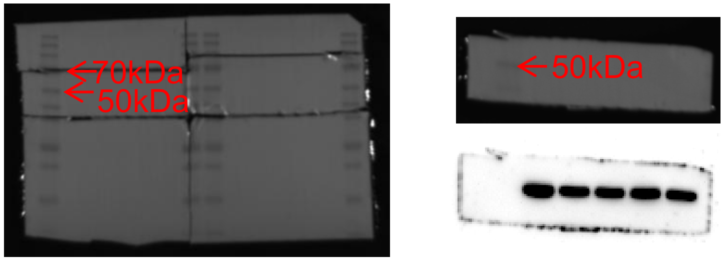

2

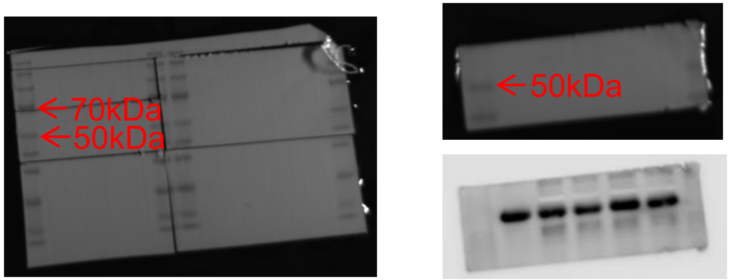

3

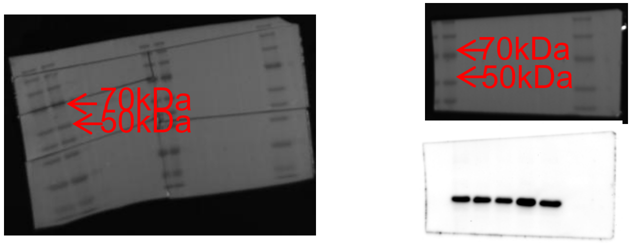

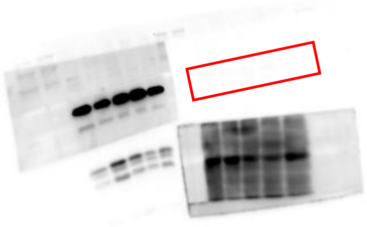

Mouse pancreatic tissue p-PI3K 55kDa

1

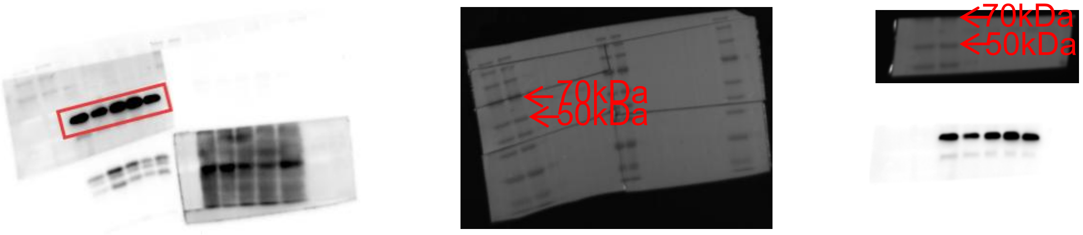

2

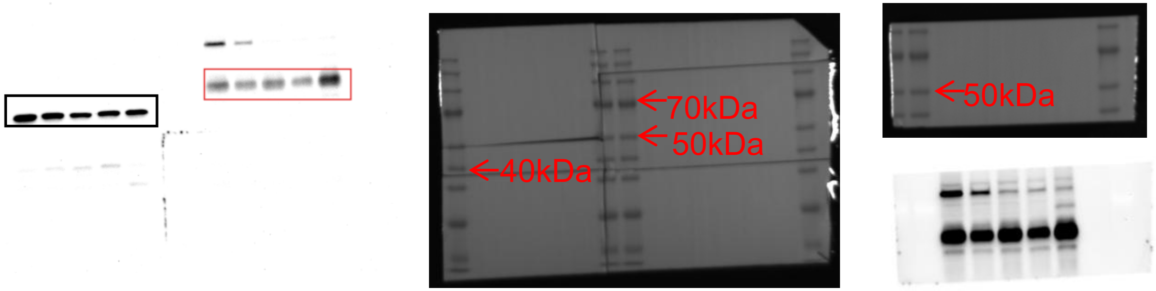

3

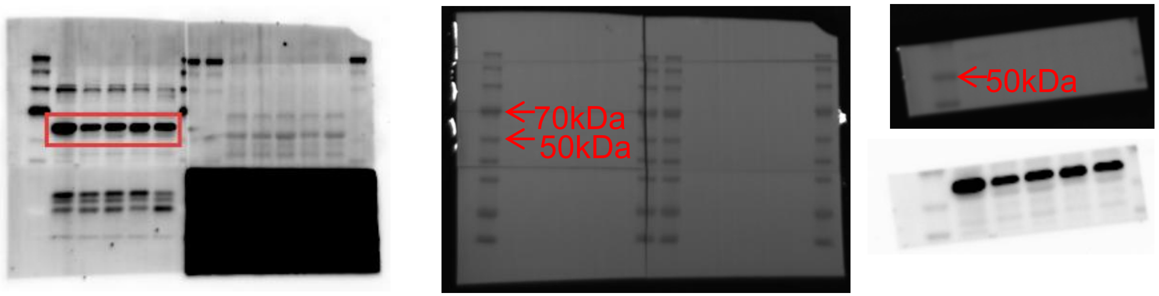

Mouse pancreatic tissue IL1β 25-35kDa

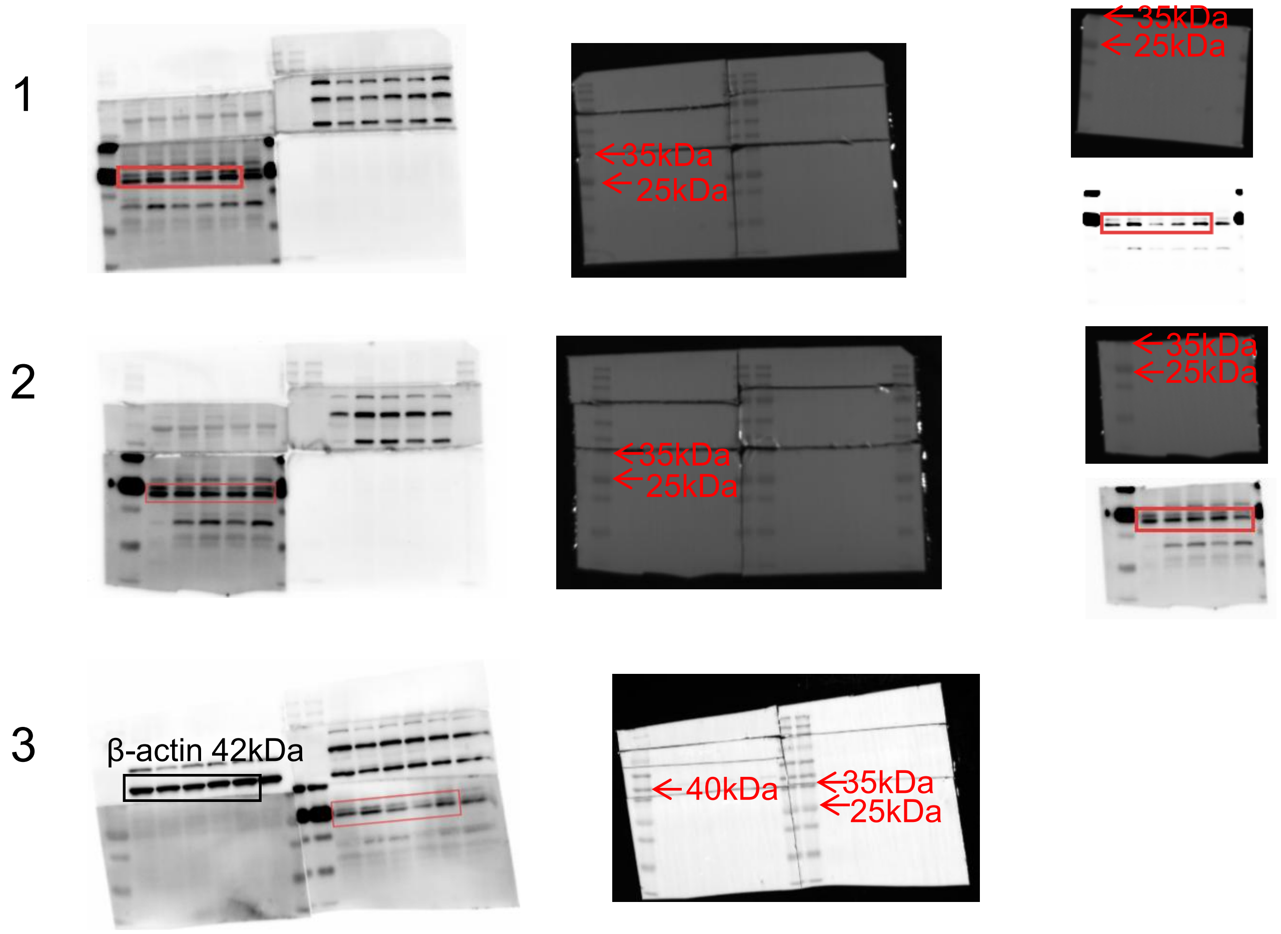

Mouse pancreatic tissue Bcl-2 26kDa

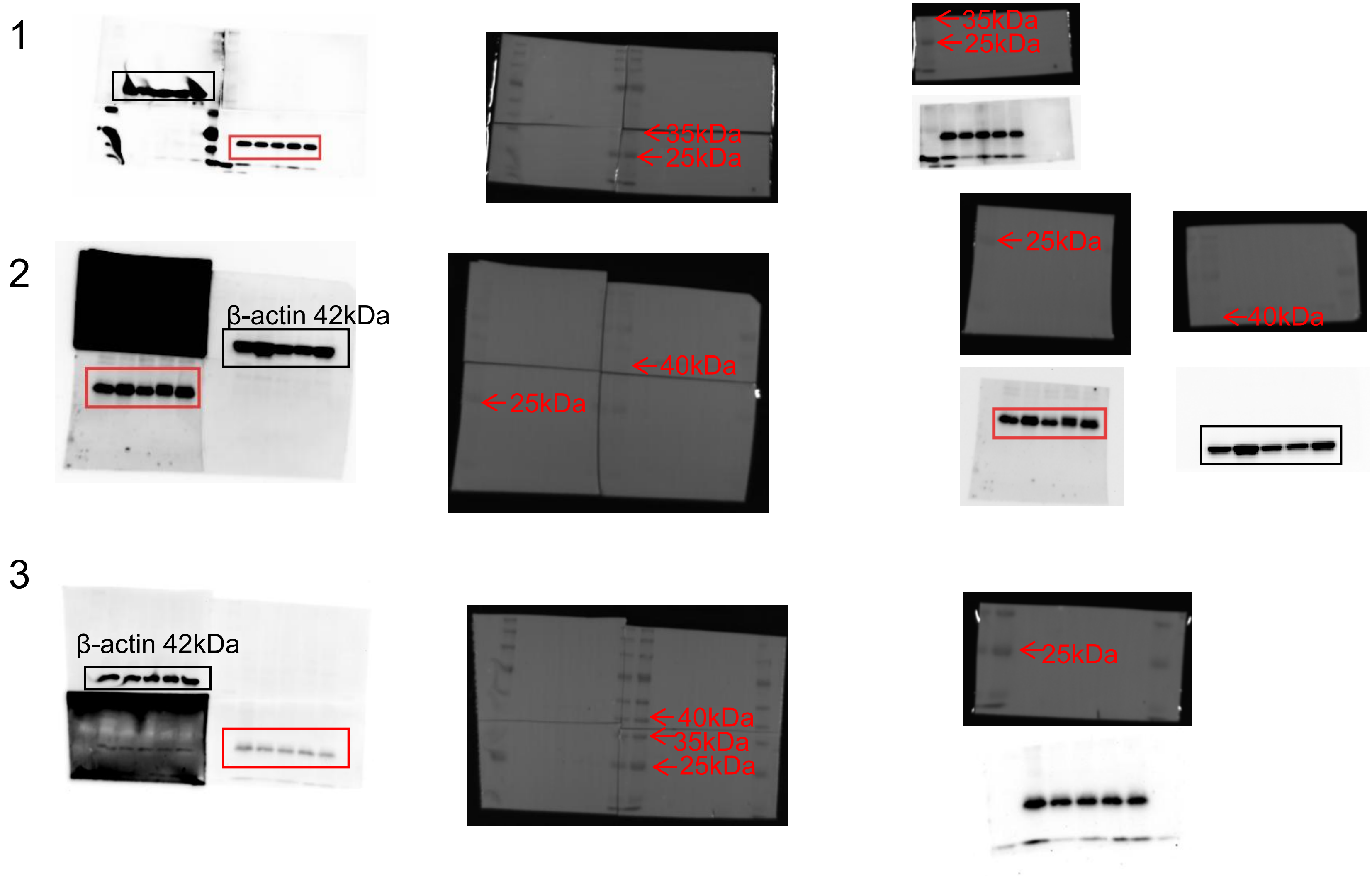

Mouse pancreatic tissue Caspase3 32kDa

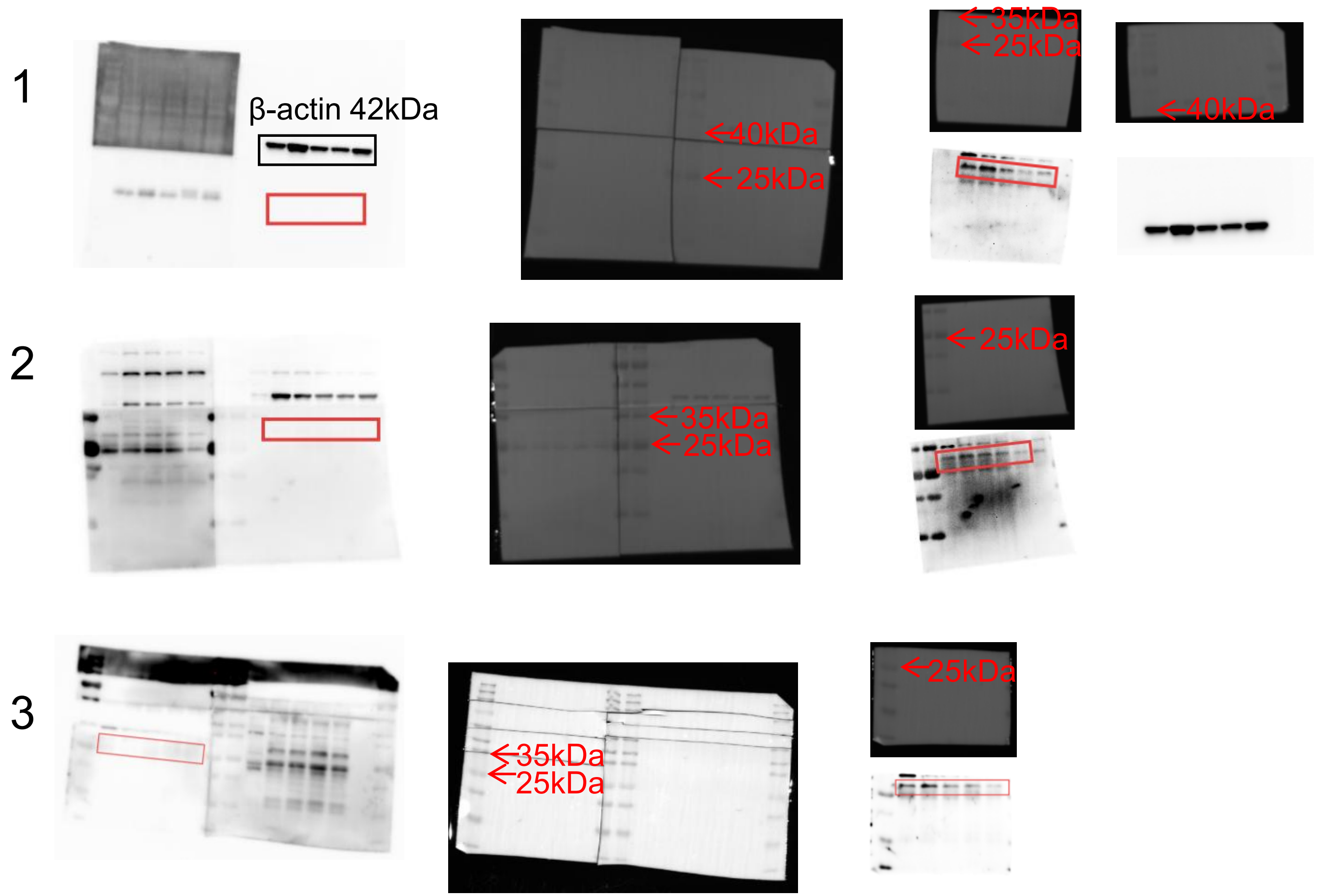

Mouse pancreatic tissue P65 65kDa

1

2

3

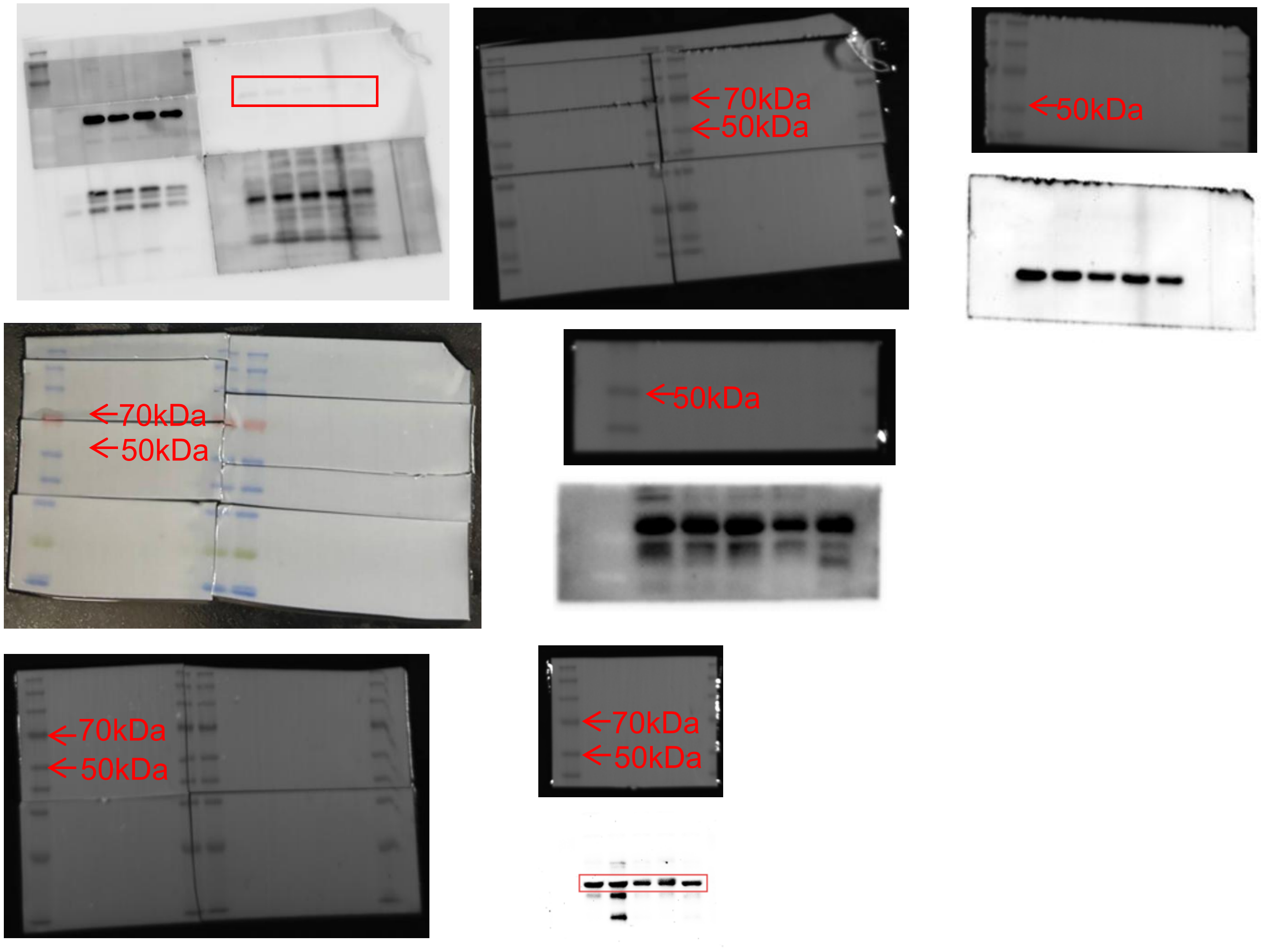

Mouse pancreatic tissue p-P65 65kDa

1

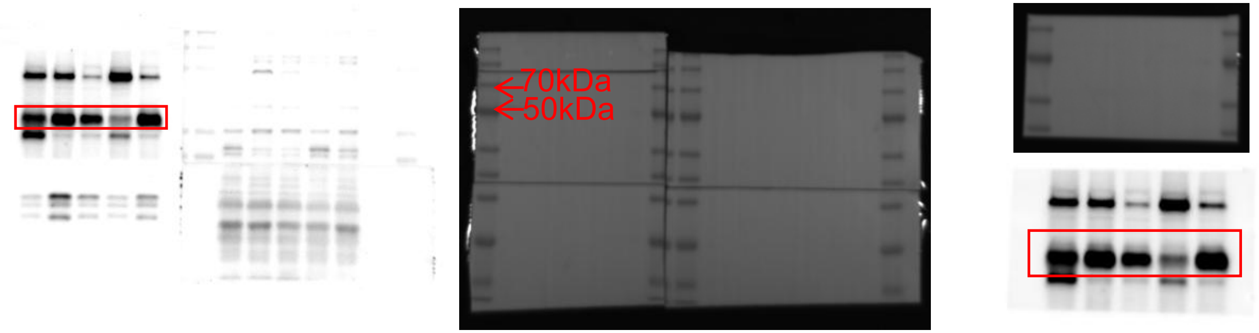

2

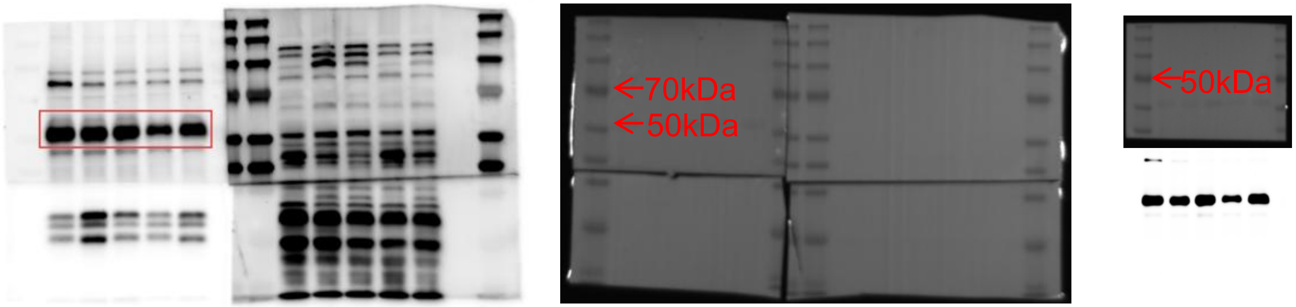

3

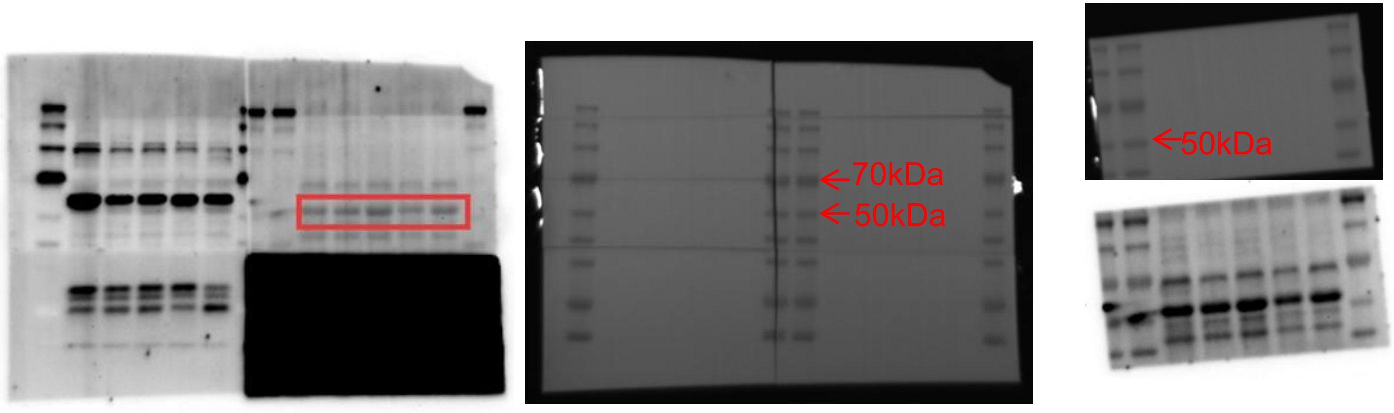

Mouse pancreatic tissue PTGER2 40-55kDa

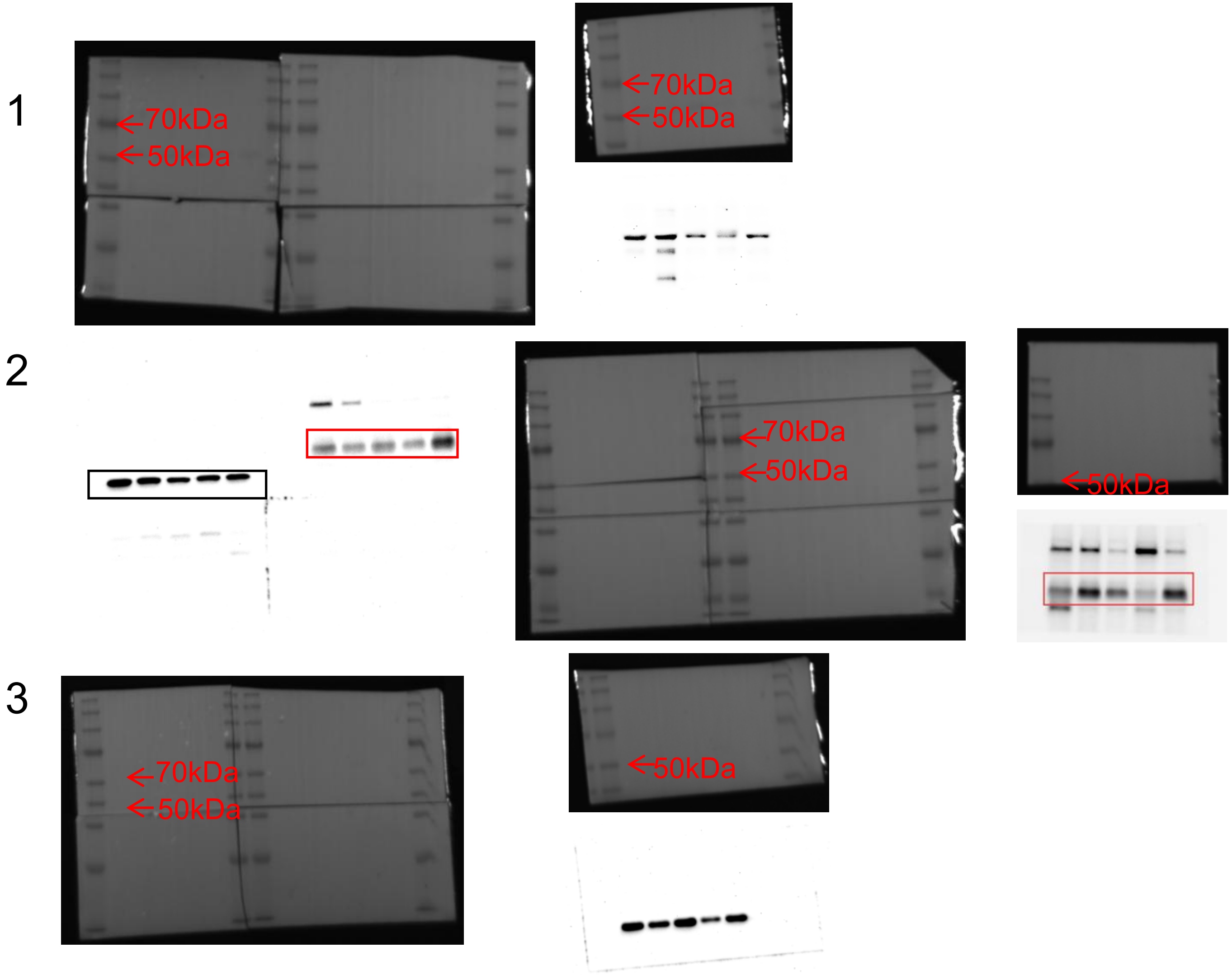

Mouse pancreatic tissue HO-1 33kDa

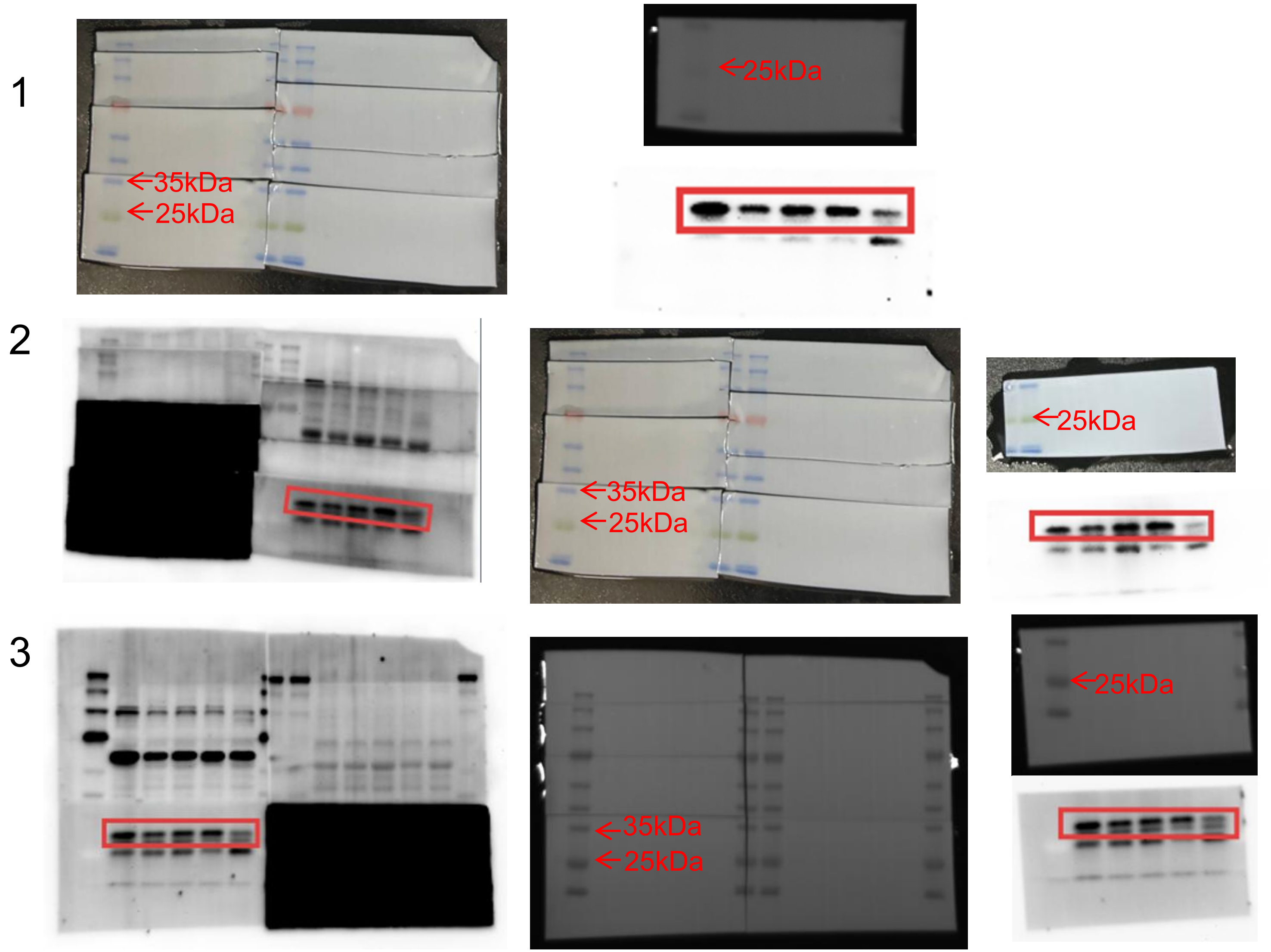

Mouse pancreatic tissue SOD-1 23kDa

1

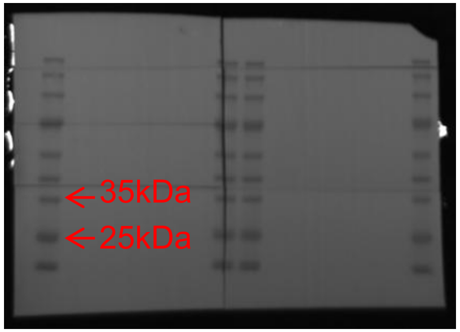

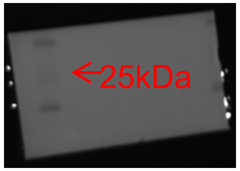

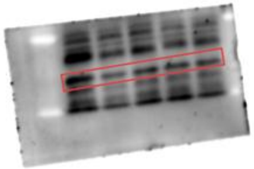

2

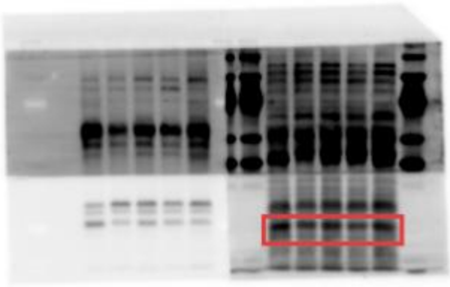

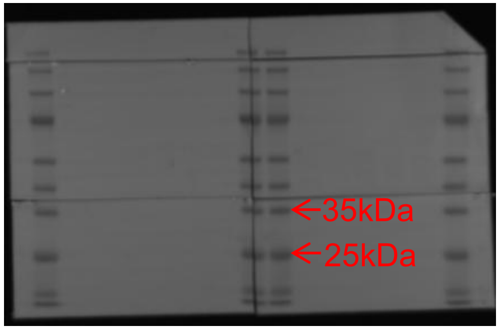

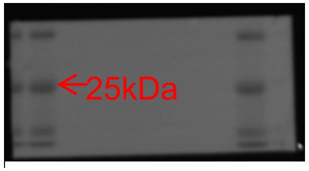

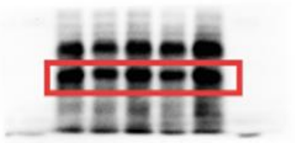

3

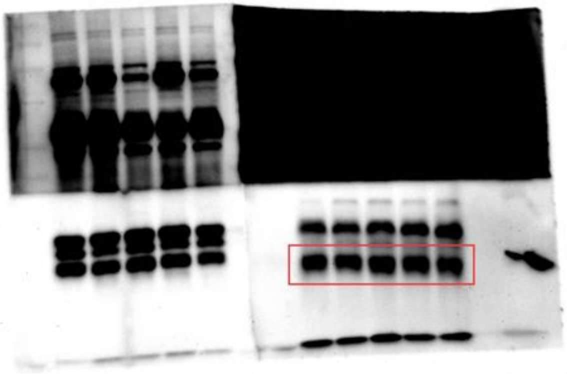

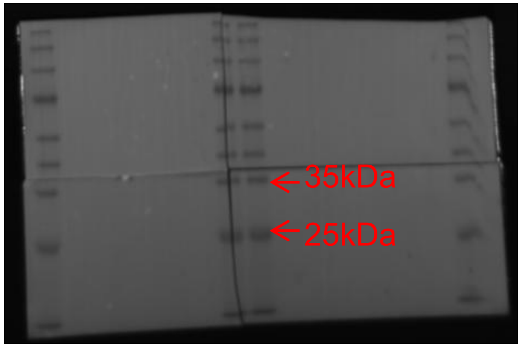

INS-1 cell AKT 56kDa

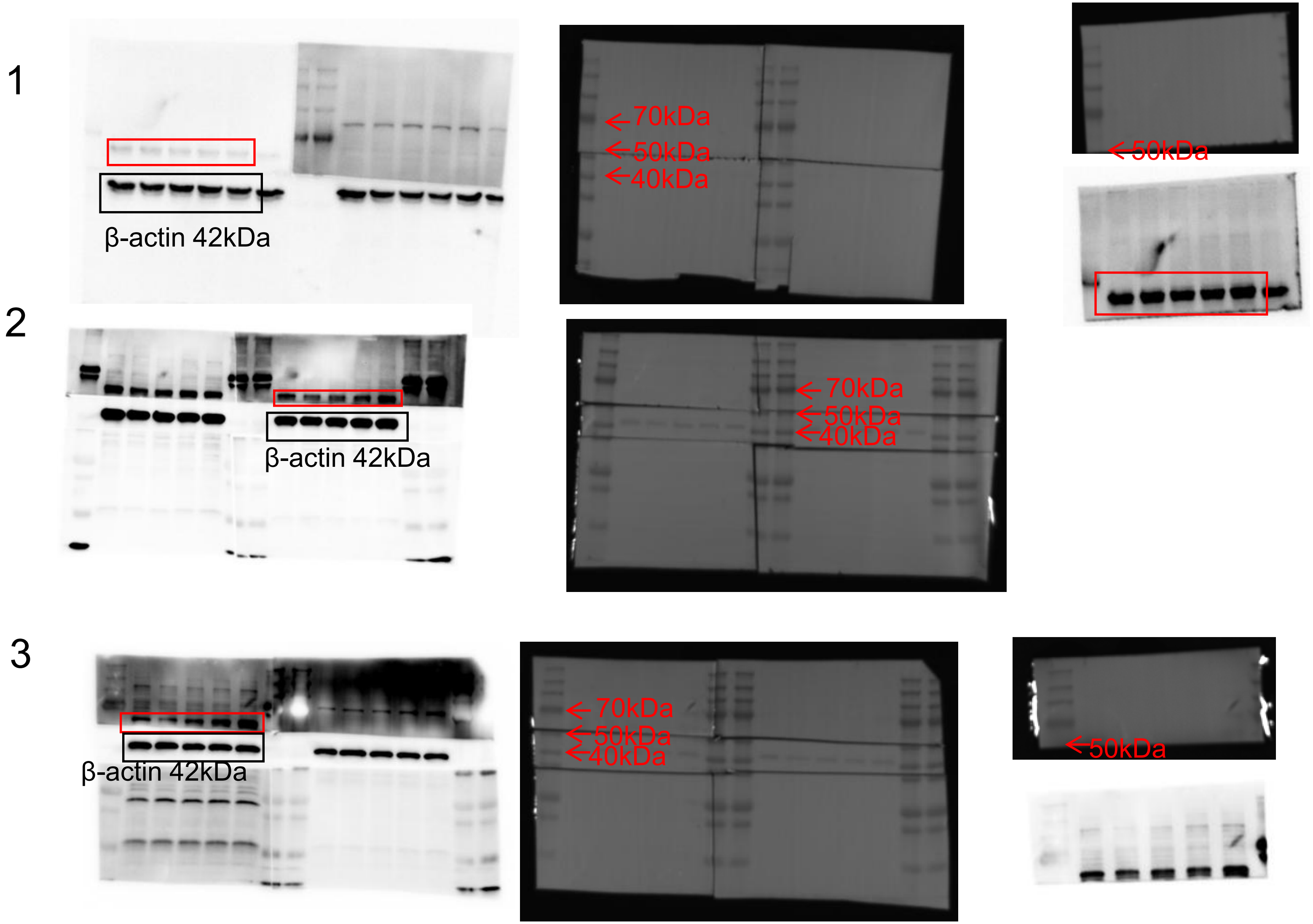

INS-1 cell p-AKT 56kDa

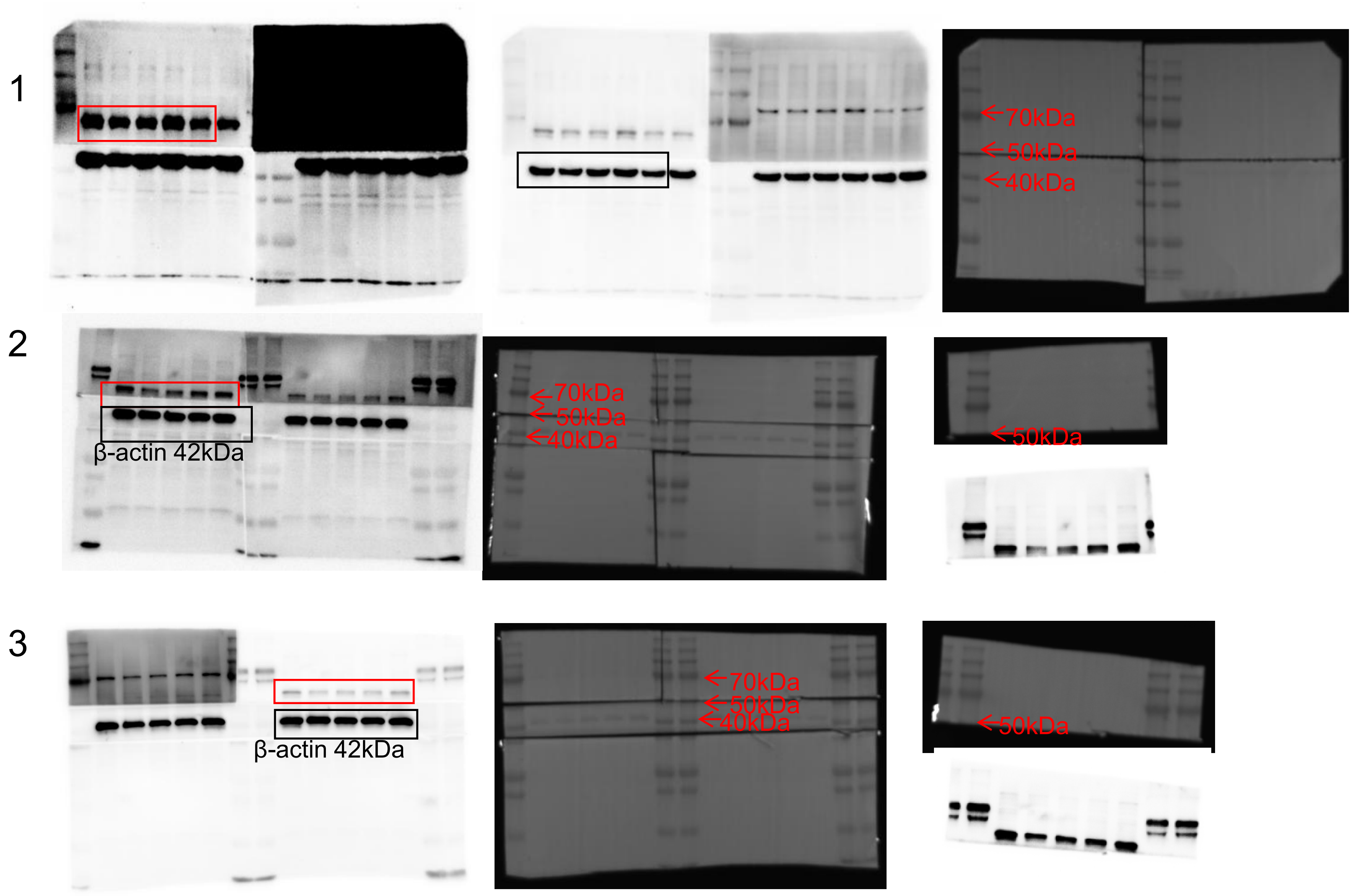

INS-1 cell PI3K 84kDa

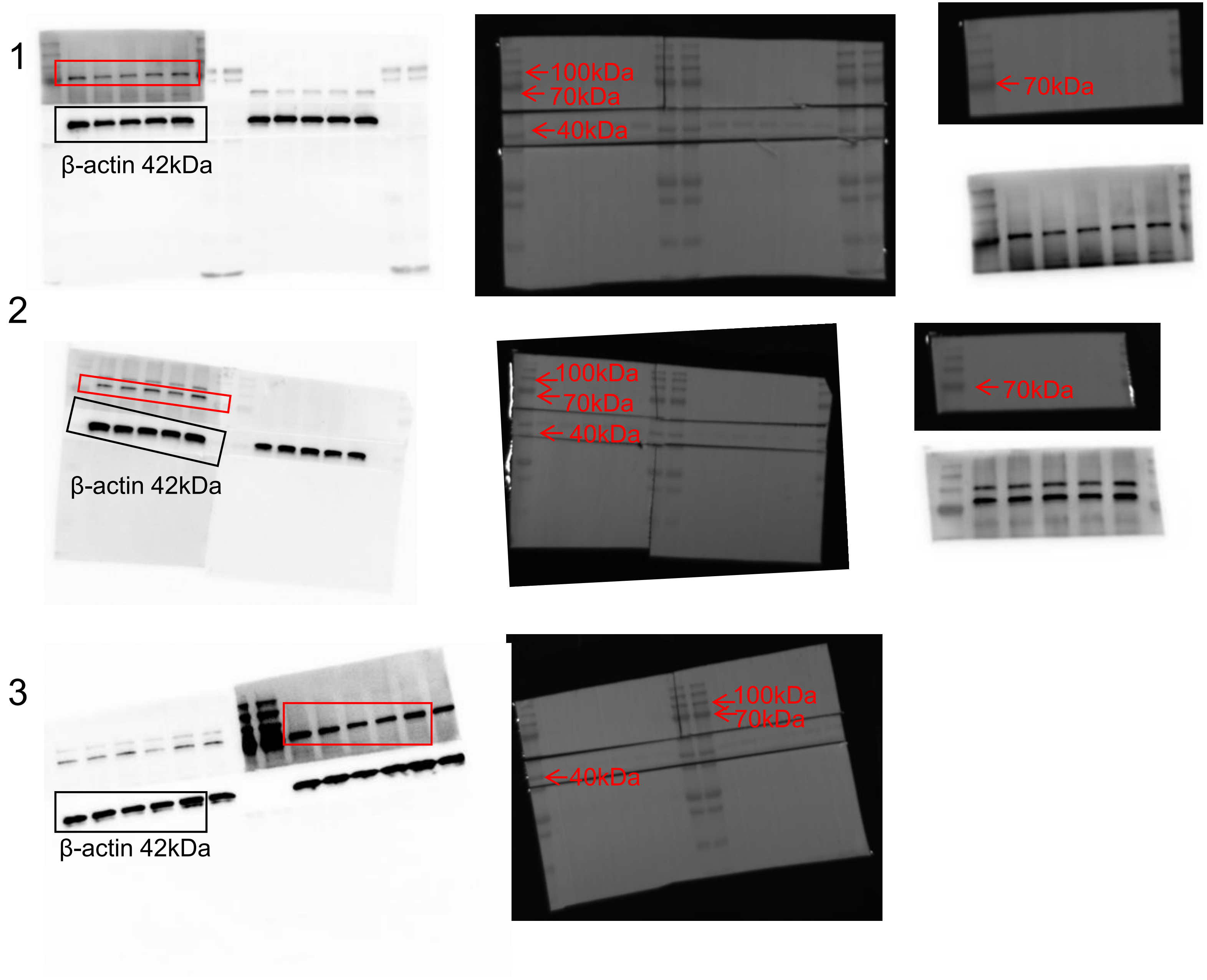

INS-1 cell p-PI3K 84kDa

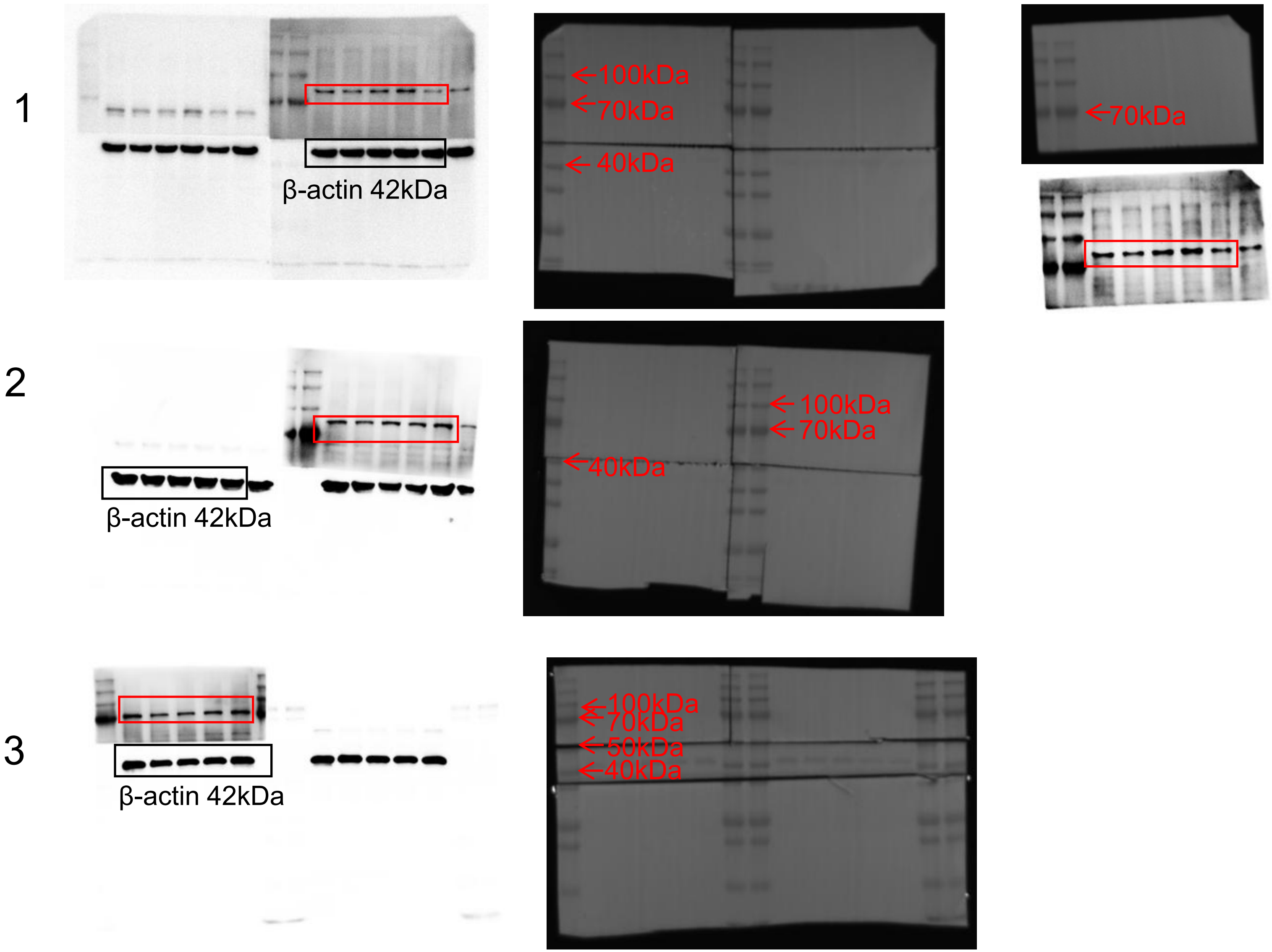

Supplement: Supplementary file 1 [file Data_Sheet_1.zip › Supplementary Materials/Supplementary File 1_Original WB blots.pdf.pdf]
